# Acidity Scale in 1,2-Difluorobenzene

**DOI:** 10.1021/acsomega.6c02089

**Published:** 2026-05-11

**Authors:** John Paulo Samin, Helerin Roomet, Märt Lõkov, Sofja Tshepelevitsh, Jaan Saame, Agnes Heering, Ivo Leito

**Affiliations:** Institute of Chemistry, 37546 University of Tartu, Ravila 14a, Tartu 50411, Estonia

## Abstract

Equilibrium Brønsted acidity measurements have been established in 1,2-difluorobenzene (1,2-DFB), both in terms of acid p*K*_a_ values measured using UV–vis spectrophotometry and unified pH (pH_abs_) values measured using differential potentiometry. The p*K*_a_ values of 137 acids were obtained in 1,2-DFB, of which 33 have been directly measured, spanning 15 orders of magnitude of acidity. The rest have been estimated using p*K*_a_ values in other solvents (acetonitrile, 1,2-dichloroethane). The p*K*_a_ values have been anchored to the computational p*K*_a_ values of 9 compounds. An experimental pH_abs_ scale spanning more than 10 orders of magnitude has been established and aligned with the aqueous pH scale, resulting in a ${pH}_{abs}^{H_{2}O}$ range from −0.6 to 10.0. The potentiometrically measured ${pH}_{abs}^{H_{2}O}$ values and ${pH}_{abs}^{H_{2}O}$ values calculated independently from the experimental p*K*_a_ values anchored to computational reference values agree well, which demonstrates the solid foundation of the underlying theory. These results open the possibility of quantitatively describing and measuring acid–base processes in 1,2-DFB, including potentiometric and UV–vis spectrophotometric ${pH}_{abs}^{H_{2}O}$ measurement.

## Graphical abstract

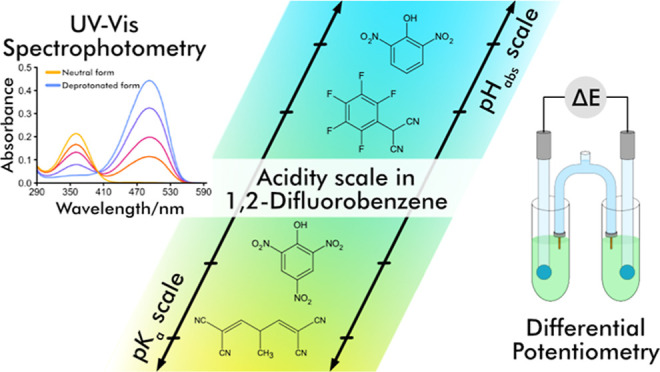

## Introduction

Solvents play a crucial role in nearly all chemical processes, including organic synthesis, electrochemistry, and metal–organic chemistry.^1^ Acids and bases take part in many, if not most, of those processes. To better understand these processes, quantitative information on the strengths of acids and bases (in terms of p*K*_a_ values), as well as the acidity of the medium (in terms of pH), is important.^2,3^

1,2-Difluorobenzene (1,2-DFB) has several useful properties that make it a valuable solvent for different applications. It is used in organic synthesis^4^ and in the study of metal–organic compounds.^5^ 1,2-DFB has also found application in lithium-ion and lithium–metal batteries, serving as an antisolvent (an active diluent that modifies solvation through solvent interactions to enhance electrolyte stability) for fluorinated electrolytes to achieve long-life battery cycles, and as a low-viscosity solvent to promote Li^+^ ion transport.^6–9^ Also, it can serve as a model for gauging solute behavior in a variety of other structurally similar solvents.^10^

Because ionic species are present in many of these processes, acid–base equilibria are important. Many of the ionic species are either deprotonated forms of acids or protonated forms of bases, such that their formation and reactivity, or in other words, the stability of these ions, are directly related to the strengths of the corresponding acids or bases. Good examples are PF_6_^–^ and (FSO_2_)_2_N^–^ anions. Both are anions of superacids. As a result, they are highly stable and have very low coordinating ability, and find use in lithium-ion batteries. PF_6_^–^ is the “standard” lithium-ion battery anion and (FSO_2_)_2_N^–^ is increasingly used as an additive anion.^11,12^ Thus, knowing the acidic and basic strengths (p*K*_a_ values) in 1,2-DFB would help to better predict and understand the processes occurring in this solvent.

1,2-DFB has very weak acidic and basic properties, making it a good differentiating solvent and suitable for measurements of compounds with a wide range of acidities. 1,2-DFB has a higher relative permittivity (ε_r_ = 13.4)^13^ than other widely used apolar solvents like toluene (ε_r_ = 2.43),^14^ 1,2-dichloroethane (ε_r_ = 10.74)^14^ and tetrahydrofuran (ε_r_ = 7.47).^14^ Consequently, 1,2-DFB is sufficiently polar to solvate neutral and charged species like organometallics and even low concentrations of inorganic salts.^5,15^

Contrary to dipolar aprotic solvents, such as DMSO^2,16,17^ or acetonitrile,^18,19^ pH and p*K*_a_ scales in nonpolar solvents are uncommon. However, in a low polarity solvent, 1,2-dichloroethane (DCE), pH^20^ and p*K*_a_ ^21^ scales have been experimentally established through relative acidity measurements of strong acids and weak bases. In tetrahydrofuran (THF), ion pair acidity scales have been constructed using Cs^+^ and Li^+^ as a counterion.^22,23^ Recently, Kong et al. have reported p*K*_a_ values in 1,2-DFB of some substituted malononitriles, fluorenes, sulfones, and ketones.^10^

The main aim of this work was to evaluate the possibility of relative p*K*_a_ measurements in 1,2-DFB, build the first self-consistent acidity scale in 1,2-DFB, both in terms of p*K*_a_ and pH_abs_ values, and compare it with similar scales in 1,2-DCE and acetonitrile. Additionally, it is of interest how well the p*K*_a_ values of acids correlate with the respective p*K*_a_ values in other solvents—first of all in MeCN and 1,2-DCE—to decide, how well values in 1,2-DFB can be predicted on the basis of data in other solvents.^24^

The acid–base equilibrium of an acid HA in a solvent S corresponds to the following equation

1 $$HA+S\overset{K_{a}}{⇄}A^{-}+{SH}^{+}$$

the acidity of acid HA in solvent S is expressed by the dissociation constant *K*_a_ of equilibrium (1)

2 $$K_{a}=\frac{a\left(A^{-}\right)\cdot a\left({SH}^{+}\right)}{a\left(HA\right)}$$

the *a* in eq 2 denotes the activity of the species in the parentheses. In most cases, the acidity is expressed as the p*K*_a_ value instead of *K*_a_

3 $$pK_{a}=-\log\left(K_{a}\right)=-\log\frac{a\left(A^{-}\right)\cdot a\left({SH}^{+}\right)}{a\left(HA\right)}$$

eqs 1–3 are strictly valid for polar solvents such as water or acetonitrile, which promote the formation of free ions. In the case of apolar solvents such as 1,2-DFB, ion pairs are formed instead of free ions, and therefore, an equilibrium between ion pairs is observed. In apolar solvents, the determination of the activity of the solvated hydrogen ion SH^+^ can be complicated. In solvents with low basicity, the dissociation of acids is limited or, depending on the solvent, even hindered to the extent that it is impossible to observe experimentally.^25,26^ To avoid the necessity to measure *a*(SH^+^) in nonaqueous media, relative acidities are often studied

4 $${HA}_{1}+A_{2}^{-}⇄{HA}_{2}+A_{1}^{-}$$

In apolar solvents like 1,2-DFB, ion pairing must be considered in eq 4

5 $${HA}_{1}+{{BH}^{+}A}_{2}^{-}⇄{HA}_{2}+{{BH}^{+}A}_{1}^{-}$$

In eq 5, BH^+^ is the counterion in the ion pair. From eq 5, the difference in ion pair acidity (Δp*K*_ip_) is defined as follows

6 $${\Delta pK}_{ip}={pK}_{ip}\left({HA}_{2}\right)-{pK}_{ip}\left({HA}_{1}\right)=\log\frac{a\left({HA}_{2}\right)\cdot a\left(BH^{+}A_{1}^{-}\right)}{a\left({HA}_{1}\right)\cdot a\left(BH^{+}A_{2}^{-}\right)}$$

If the Δp*K*_ip_ value is corrected using the difference of ion pair dissociation constant Δp*K*_d_, then the Δp*K*_a_ values can be obtained as shown in eq 7.

7 $$\Delta pK_{a}=\Delta pK_{ip}-\log\frac{K_{d}\left({BH}^{+}A_{2}^{-}\right)}{K_{d}\left({BH}^{+}A_{1}^{-}\right)}=\Delta pK_{ip}+\Delta pK_{d}$$

The conventional pH scale is based on the activity of H^+^ ions in water.^27^ Reliable measurement of pH in nonaqueous solvents is difficult, because H^+^ ions are solvated differently depending on the medium, meaning that every solvent has its own individual pH scale, and pH scales of different solvents cannot be directly compared.^28,29^ The introduction of the unified pH (pH_abs_) approach^30^ could address this issue of noncomparability between solvents. In the pH_abs_ scale, an ideal proton (hydrogen ion) in the gas phase at standard state was used as a reference point in all media. Here, the absolute chemical potential of the proton is arbitrarily set to 0 kJ mol^–1^, and when the proton interacts with any medium, it is stabilized, thereby decreasing its chemical potential and consequently increasing the pH_abs_ of the system.^30^ However, it is impossible to make direct measurements against this reference point. Instead, a more suitable reference is required, which the conventional aqueous pH scale can provide because of its convenience and experimental accessibility.^20^ Thus, pH_abs_ values are typically expressed as “aligned to the conventional aqueous pH scale”, expressed as ${pH}_{abs}^{H_{2}O}$ . The ${pH}_{abs}^{H_{2}O}$ values measured in any solvent are directly comparable to the pH values of the conventional aqueous pH scale.

Unified pH_abs_ measurements can be performed through differential potentiometry. In this method, the potential difference between two electrodes submerged in two different solutions is measured. Relative acidities (ΔpH_abs_) can then be obtained from these potential differences using the mean slope of the electrodes determined during calibration against a reference electrode in aqueous standard buffers.

8 $$\Delta pH_{abs}=\frac{\Delta E}{{slope}_{mean}}$$

Furthermore, linking these pH_abs_ values to the aqueous pH scale can now be achieved by comparing the pH_abs_ of the solutions to the pH of the aqueous standard buffer solutions. The new values are represented as ${pH}_{abs}^{H_{2}O}$ , indicating that they use aqueous pH values as the reference points, and the comparison of pH values measured in different solvents is now possible.

## Experimental Section

### Chemicals

The solvent, 1,2-difluorobenzene (1,2-DFB) with purity of 98% and analytical grade (Fluorochem Ltd.), was used in this study. The solvent was distilled prior to use, and the distilled solvent was dried using 3 Å molecular sieves (Waco Inc.) for at least 24 h. Its water content was regularly checked using a coulometric Karl Fischer titrator (Mettler Toledo DL 32), ensuring water concentration remained between 1 and 5 ppm, which is considered sufficiently low for this work. (*Tert*-butylimino)­tris­(pyrrolidino)­phosphorane (*t*BuP_1_(pyrr)_3_, Fluka, 97%), triflic acid (TfOH, Aldrich, 99%), bis­(trifluoromethane)­sulfonimide (99%), **1** (Reakhim, high purity), **2** (Alfa Aesar, 98%), **14** (Reakhim, analytical standard), saccharin (Aldrich, ≥99%), pentabromophenol (Aldrich, ≥95%), (3*Z*)-3-methylpenta-1,3-diene-1,1,5,5-tetracarbonitrile (**25**, in tetraethylammonium Et_4_N^+^ salt form, Aurora Fine Chemicals, ≥95%), penta-1,3-diene-1,1,5,5-tetracarbonitrile (**28**, in tetraethylammonium Et_4_N^+^ salt form, Aurora Fine Chemicals, ≥95%), ammonium formate (Aldrich, ≥99%), ethanol (Honeywell, absolute ≥99.8%), acetonitrile (Romil, ≥99%), the ionic liquid, triethylpentylammonium bis­(trifluoromethanesulfonyl)­imide ([N_2225_]­[NTf_2_], Iolitec, 99%) were obtained commercially. All remaining compounds have been previously used in various publications: **3**, **5**, **6**, **8**, **10–12**, **17**, **27**, **30**;^26^ **4**;^19^ **40**;^20^ **7**, **13**;^31^ **9**;^21^ **15**, **16**, **19**, **20**;^32^ **29**, **31**–**33**;^33^ **18**, **22**;^34^ **21**, **23**, **24**, **26**;.^35^

### p*K*_a_ Determination in 1,2-DFB

#### Background

The acidity of compounds in 1,2-DFB was characterized by determining their p*K*_a_ values using UV–vis spectrometry. The acidity of a compound can be assessed by titrating the solution and recording its UV–vis spectra. These spectra give the relative amounts of the neutral and anionic species in the solution, which could be used to calculate an absolute p*K*_a_ of the studied acid, if the activity of the solvated proton in the solvent (in absolute terms, i.e., using the protonated solvent as standard state) is known. Because the activity of the solvated proton is difficult to measure in nonaqueous systems such as 1,2-DFB, a relative method is applied instead: a mixture of two acids is titrated, and the observed spectral changes allow the difference of their p*K*_a_ values (Δp*K*_a_) to be determined. Absolute p*K*_a_ values can still be obtained if one of the acids has an independently determined p*K*_a_ and serves as the reference. This is also done in this work, by using computational p*K*_a_ values of several acids as reference p*K*_a_ values, as described below.

#### Method

The Δp*K*_a_ values were determined using essentially the same methodology as for Δp*K*_a_ measurements in acetonitrile and described in detail in previous publications^36,37^ with one important difference: due to the lower dielectric permittivity of 1,2-DFB (13.4 ^13^) ion pairing cannot be neglected. Thus, the directly determined quantities are the Δp*K*_ip_ values (differences of ion pair acidities) corresponding to eq 6, which are thereafter corrected for ion pairing via eq 7 using the Δp*K*_d_ estimated from the Fuoss equation (as described in ref 21) to obtain the Δp*K*_a_ values. A brief description follows.

The Δp*K*_ip_ determination between two acids consists of three steps. The first two steps involve the separate titrations of two acids for which the Δp*K*_ip_ value is determined, using both basic and acidic titrants to obtain the UV–vis spectra of the neutral and protonated forms of these compounds. The titration is carried out by stepwise addition of the titrant using Hamilton gastight syringes. After each addition of the titrant, the UV–vis spectrum is measured. As the third step, a 1,2-DFB solution containing both acids is similarly titrated to obtain 15–30 UV–vis spectra, including those of the acids in their neutral forms and fully deprotonated forms, as well as a number of spectra where both acids are partially deprotonated. The obtained spectral data was mathematically treated at multiple wavelengths using multilinear regression analysis in MS Excel. This treatment yielded the dissociation levels (α) of both acids in all the mixtures formed during the titration of both acids together in the same solution

9 $$\alpha =\frac{\left[A^{-}\right]}{\left[A^{-}\right]+\left[HA\right]}$$

According to the Hammett assumption,^38^ the activity coefficient ratios of the anions and their corresponding neutral forms in a given solution are the same for all acids. This assumption holds very well if the solvent is the same and the acids and their anions are not too different by their molecular size and the extent of charge delocalization in their anions, which is true for the acids for which Δp*K*_ip_ values were measured in this work. Then, the activity ratios in eq 6 can be rewritten as ratios of the species’ equilibrium concentrations in the solution. These ratios are directly related to the degrees of dissociation (α) of the acids, which were used to calculate the differences in p*K*_ip_ values (Δp*K*_ip_) according to the following equation

10 $$\Delta {pK}_{ip}=\log\frac{\alpha_{1}\left(1-\alpha_{2}\right)}{\alpha_{2}\left(1-\alpha_{1}\right)}$$

The multilinear regression calculation method is described in more detail in the Supporting Information of a previous publication.^37^ Every Δp*K*_ip_ value determined this way corresponds to one arrow in Table 1.

**Table 1:**
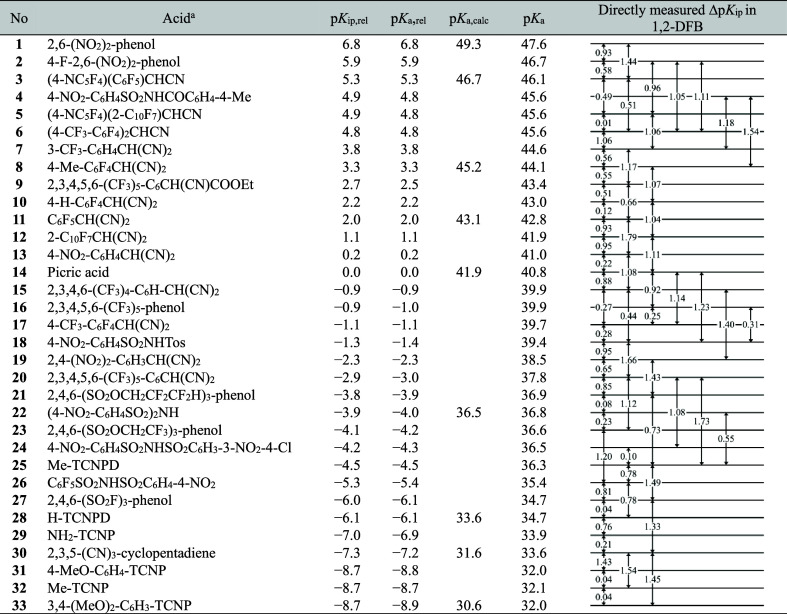
Experimental p*K*_a_ Scale in 1,2-DFB

a Tos represents the 4-Me-C_6_H_4_SO_2_–group; TCNPD—1,1,5,5-tetracyanopentadiene; TCNP—1,1,3,3-tetracyanopropene.

To obtain Δp*K*_a_ values from the experimentally measured Δp*K*_ip_ values, the Δp*K*_ip_ values were corrected with the difference of ion pair dissociation constants (Δp*K*_d_) of each acid pair obtained using a formula derived from the Fuoss model.^39^ The same approach has been previously used in the apolar solvent 1,2-DCE by Paenurk et al.^21^

According to the Fuoss model, the ion pair dissociation constant *K*_d_ can be expressed as follows

11 $$K_{d}=\frac{{3000\cdot e}^{b}}{{4\cdot \pi \cdot N\cdot a}^{3}}$$

in eq 11, *N* is the Avogadro constant (6.02 × 10^23^ mol^–1^), *a* is the interion distance (equal to the sum of *r*_A_^–^ and *r*_B_^+^ in cm), and *b* is expressed as follows

12 $$b=\frac{{-q}^{2}}{{a\cdot \varepsilon }_{r}\cdot k\cdot T}$$

in eq 12, *q* is the elementary charge (4.80 × 10^–10^ cm^3/2^ g^1/2^ s^–1^), *k* is the Boltzmann constant (1.38 × 10^–16^ cm^2^ g s^–2^ K^–1^), and *T* is the temperature. eq 11 can be rewritten in the following way

13 $${pK}_{d}=-\log\frac{3000}{4\cdot \pi \cdot N}+\frac{q^{2}\cdot \log e}{\varepsilon_{r}\cdot k\cdot T}\times \frac{1}{a}+3\log a$$

To express the Δp*K*_d_ of a pair of acids HA_1_ and HA_2_, eq 13 is rewritten

14 $$\Delta pK_{d}=C\left(\frac{1}{r_{A_{2}^{-}}+r_{B^{+}}}-\frac{1}{r_{A_{1}^{-}}+r_{B^{+}}}\right)+3\log\left(\frac{r_{A_{2}^{-}}+r_{B^{+}}}{r_{A_{1}^{-}}+r_{B^{+}}}\right)$$

in eq 14, *r*_A1_^–^ and *r*_A2_^–^ are the ionic radii of the anions of acids HA_1_ and HA_2_, *r*_B+_ is the ionic radius of cation B^+^ taking part in the ion pair formation, and *C* is a constant (*C* = *q* ^2^ × log *e* × ε_*r*_^–1^ × *k* ^–1^ × *T* ^–1^).

The ionic radii used in this work were the average of the three radii found using three methods: (1) calculation from molecular volume using sphere volume formula, (2) derivation from the dimension of an imaginary cuboid inside which the ion’s molecular surface would exactly fit, and (3) average distance of surface segments from the ion’s centroid (as listed in the COSMO file).

The Fuoss model assumes spherical ions with uniformly distributed (i.e., well delocalized) charge. The charge in both the anions and cations of this work can be considered sufficiently delocalized and the phosphazenium cations are approximately spherical. However, the tetraalkylammonium ions (counterions for some acid anions in the used salts) and most acid anions cannot be considered spherical. This leads to some over- or undercompensation of the ion-pairing effect. However, because (1) low concentrations were used and (2) the measurements were relative, the whole effect of ion pairing, i.e. difference between p*K*_ip_ and p*K*_a_ values, amounts to at most 0.2 p*K*_a_ units (in majority of cases less than 0.1 p*K*_a_ units). So considering the small magnitude of the correction, slight over- or undercompensation is not a problem. It is important to add that such over- or undercompensation only shifts the p*K*_a_ value of the respective compound in the scale but does not cause contraction or expansion of the scale.

#### Instruments and Chemicals

All Δp*K*_ip_ determinations were carried out inside an MBraun Unilab glovebox filled with argon (5.0, Linde Gas) to avoid the effects of moisture and oxygen. The moisture and oxygen contents inside the glovebox were constantly monitored and were always under 10 ppm. An Agilent Cary 60 spectrophotometer, connected via optical fiber cables to an external cell compartment located inside the glovebox, was used for the spectrophotometric titrations. A scanning speed of 600 nm/min was used to record the UV–vis spectra. This setup enabled measurements to be carried out entirely within the glovebox. The spectrometer was operated via Cary WinUV 5.1 program, data processing was done using Microsoft Excel. In this work, the p*K*_a_ measurements were performed at temperature of 24.1 ± 0.7 °C.

Triflic acid (Aldrich, ≥99%) was used to prepare the acidic titrant solution and *tert*-butylimino-tris­(pyrrolidino)­phosphorane (Aldrich, ≥97%) was used to prepare the basic titrant solution. The concentrations of the acidic titrant solutions were (1.6 × 10^–3^ – 1 × 10^–2^) mol L^–1^ and the concentrations of the basic titrant solutions were (1.7 × 10^–3^ – 5.1 × 10^–2^) mol L^–1^. These titrants were strong enough to either deprotonate the acids studied or protonate their anions. Moreover, they were sufficiently stable and did not absorb UV–vis radiation during the measurements, which makes them suitable for the study. The concentrations of the studied acids were (4.8 × 10^–6^ – 1.5 × 10^–4^) mol L^–1^ during the titrations.

### pH_abs_ Determination in 1,2-DFB

The determination of pH_abs_ values was carried out in equimolar buffer solutions of the acids and their salts with protonated *t*BuP_1_(pyrr)_3_ in 1,2-DFB. The measurements were performed using differential potentiometry in a manner similar to that described previously.^20,40,41^ This method measures the potential difference between two glass electrodes (GE) immersed in separate solutions (connected by a salt bridge), thereby providing the relative pH differences between them.

Equimolar buffer solutions were prepared for each compound in 1,2-DFB, where the acid and its corresponding anion concentrations are approximately equal (∼0.333 mM). An appropriate amount of each compound was weighed into a 4 mL glass vial. The compound was then dissolved by adding 3 mL 1,2-DFB to the vial. The contents of the vial were transferred to a 100 mL reagent bottle. The vial was rinsed four more times with 3 mL portions of 1,2-DFB, resulting in a cumulative volume of 15 mL. Approximately 4.17 mg of *t*BuP_1_(pyrr)_3_ and 3.75 mg of bistriflimide specifically for Me-TCNPD (**25**) were weighed in a separate 4 mL glass vial and dissolved in a similar manner with the compound using 3 mL of 1,2-DFB. Its contents were also transferred to the same reagent bottle. To bring the concentration of the prepared buffer solution to 0.333 mM, an additional 10 mL 1,2-DFB was added to the reagent bottle. All compounds were mixed with phosphazene base, except for Me-TCNPD (**25**), which was combined with bistriflimide to prepare the buffer solution. All compounds used in the measurements were fully dissolved in 1,2-DFB. For compounds that required extended time for full dissolution, solutions initially containing partly undissolved salt were left in the glovebox for 24–48 h to ensure complete dissolution before use.

The differential potentiometric setup consists of metal solid contact glass electrodes (ECT-0601, Izmeritelnaya tekhnika, Moscow, Russia) and a glass salt bridge filled with [N_2225_]­[NTf_2_] with PEEK capillary tubes (I.D. 0.13 mm) at the ends to prevent leakage and ensure a very slow release of the ionic liquid to the half cells shown in Figure 1. The differential potentiometric cell was configured as

$$GE2\left|Solution2∥\left[N_{2225}\right]\left[N{Tf}_{2}\right]\right|\left|Solution1\right|GE1$$

where GE 1 and GE 2 are close to identical metal solid contact glass electrodes, and [N_2225_]­[NTf_2_] serves as the ionic liquid salt bridge electrolyte between the two solutions. This ionic liquid was suitable for the setup because its cation and anion have nearly identical diffusion and solvation properties, effectively suppressing or nearly canceling the liquid-junction potential across the system.^42^

**Figure 1:**
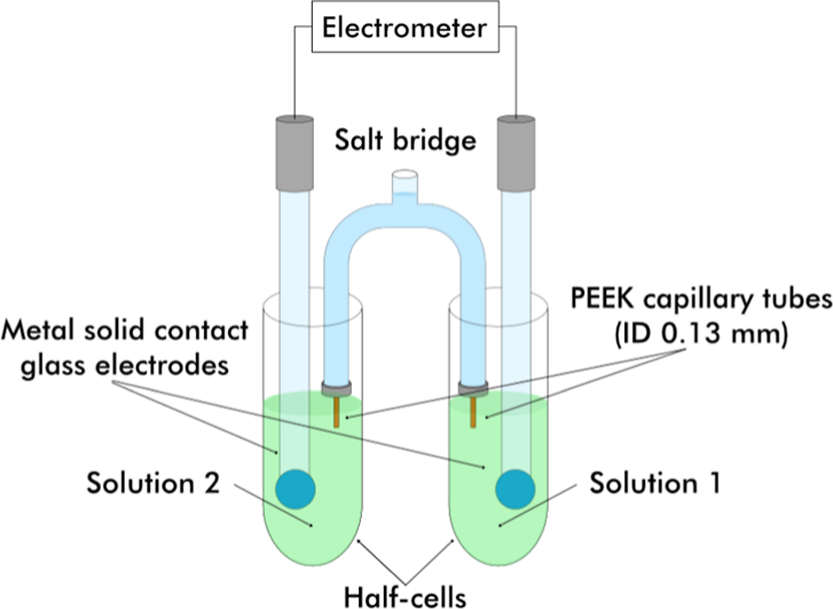
Diagram of the electrochemical setup.

Before the measurements, the electrodes were immersed in 1,2-DFB for stabilization, after which approximately 10 mL of sample solution was added to each half-cell and the electrodes are carefully immersed in the solution. The setup was enclosed inside a Faraday cage to prevent electromagnetic interference during the measurements. The potential was measured using an electrometer/high resistance meter (Keysight B2987A, California, USA). The potential readings were recorded at an interval of 10 s for at least an hour or as necessary, to observe stable readings. The data was acquired using the Quick IV Measurement software. The data points with the most stable 15-min measurements were selected, averaged and used for the calculation of the ΔpH_abs_ values in 1,2-DFB. The mean slope −58.35 mV was used for the calculation of ΔpH_abs_ values. Every arrow in Table 6 represents the average ΔpH_abs_ from one such measurement series. The results were deemed acceptable only if the standard deviation of the 15 min measurement was below 1 mV and the observed drift was less than 4 mV h^–1^.

Furthermore, all steps from preparing solutions in 1,2-DFB to conducting potentiometric measurements were carried out inside a glovebox with an argon atmosphere to prevent moisture interference. The glovebox was maintained at a temperature of 25.0 ± 0.2 °C and moisture content of less than 0.1 ppm.

### Compiling the p*K*_a_ and pH_abs_ Scales in 1,2-DFB

The assigned p*K*_a_ and pH_abs_ values of compounds in 1,2-DFB were obtained using the “ladder approach”.^20,40^ This is performed through least-squares minimization by minimizing the sum of squares (SS) of the differences of experimentally determined Δp*K*_ip_ or ΔpH_abs_ values over all the measurements and the assigned p*K*_a_ or pH_abs_ values while keeping the p*K*_a_ or pH_abs_ value of the anchor point constant

$$SS=\overset{n_{m}}{\underset{i=1}{\sum }}{\left\{{\Delta pK}_{ip}^{i}-\left[{pK}_{a}\left({HA}_{2}\right)-{pK}_{a}\left({HA}_{1}\right)\right]\right\}}^{2}for\;pK_{a}scale$$ 15a

15b $$SS=\sum_{i=1}^{n_{m}}{\left\{{\Delta pH}_{abs}^{i}-\left[{pH}_{abs}\left({HA}_{2}\right)-{pH}_{abs}\left({HA}_{1}\right)\right]\right\}}^{2}\text{for}\;{pH}_{abs}scale$$

The reliability and consistency of the acidity scales can be evaluated by using the consistency standard deviation (consistency parameter), which is defined by the following equation

16 $$s=\sqrt{\frac{SS}{n_{m}-n_{c}}}$$

in eq 15a,b, *n*_m_ is the number of Δp*K*_ip_ or ΔpH_abs_ measurements, and *n*_c_ is the number of assigned p*K*_a_ or pH_abs_ values. The consistency parameter, s, is the estimate of a part of the average standard uncertainty (with coverage probability of approximately 68%, also called “one sigma” uncertainty) of the values on the scale. It accounts for all random effects and intraday systematic effects in Δp*K*_ip_ or ΔpH_abs_ measurements. In the case of the used measurement methodology, systematic effects can occur, which bias all Δp*K*_ip_ or ΔpH_abs_ measurement results in the same direction (causing a decrease or increase in the range of the scale) or shift a specific compound on the scale in relation to other compounds. These effects are not accounted for by the consistency parameter. Being an average parameter, it also neglects that the quality of the individual Δp*K*_ip_ or ΔpH_abs_ measurements can vary between different compounds and that the reliability of the p*K*_a_ or pH_abs_ value depends on how far from the anchor point(s) the value of a particular compound lies.^26^ Importantly, the consistency parameter does not consider the uncertainty of anchoring the p*K*_a_ scale, which, in the case of anchoring to computational values, is very large. Because of these reasons, the consistency standard deviation, as calculated in eq 16, is strictly speaking not interpretable as the overall combined standard measurement uncertainty of individual p*K*_a_ or pH_abs_ values. However, this parameter provides a reasonable estimate of the overall quality of the measurements, is useful as an uncertainty estimate when comparing values that are all from the same scale, and allows us to compare the reliability of p*K*_a_ or pH_abs_ scales in different solvents.

The p*K*_a_ values of compounds in 1,2-DFB were obtained by anchoring the relative p*K*_a_ values (p*K*_a,rel_) to the computational p*K*_a_ (p*K*_a,calc_) values of nine compounds (Table 1): 2,6-(NO_4_)_2_-phenol (**1**), (4-NC_5_F_5_)­(C_6_F_5_)­CHCN (**3**), 4-Me-C_6_F_4_-CH­(CN)_2_ (**8**), C_6_F_5_CH­(CN)_2_ (**11**), picric acid (**14**), (4-NO_2_-C_6_H_4_-SO_2_)_2_NH (**22**), H-TCNPD (**28**), 2,3,5-(CN)_3_-cyclopentadiene (**30**), and 3,4-(MeO)_2_-C_6_H_3_-tetracyanopropene (**33**). The p*K*_a_ values of multiple compounds were calculated and used as anchors to reduce the effect of random error of a computational anchor value on the results. Least square minimization was used to determine an offset that was added to the scale to minimize the sum of squared differences between the calculated and experimental p*K*_a_ values. Furthermore, the correlations between 1,2-DFB and other solvents (MeCN and 1,2-DCE) were also investigated.

For the pH_abs_ values of the compounds in 1,2-DFB to be comparable to the conventional pH scale, bridging solutions, 10 mM ammonium formate solution in absolute ethanol ( ${pH}_{abs}^{H_{2}O}$ = 8.9) and acetonitrile/pH 4 formate (60/40 v/v) solution ( ${pH}_{abs}^{H_{2}O}$ = 6.0), were used as anchoring points. This establishes a reliable connection between the pH values of 1,2-DFB and the conventional aqueous pH scale. Bridging solutions with known ${pH}_{abs}^{H_{2}O}$ values were used because direct comparison of solutions in 1,2-DFB with standard aqueous buffer solutions is challenging (even trace amounts of water can significantly alter their pH).

### Computational p*K*_a_ Values of “Anchor” Acids in 1,2-DFB

The p*K*_a_ values of 9 acids in 1,2-DFB were computed by combining their gas-phase dissociation Gibbs energies and solvation Gibbs energies, similarly to the method used in ref 21 (methodology is explained in detail in the Supporting Information). The solvation Gibbs energy of the proton in 1,2-DFB, Δ_solv_ *G*°(H^+^) = −899 kJ mol^–1^, was computed by Scholz et al.^43^ using the rCCC model.^44^ This Δ_solv_ *G*°(H^+^) value corresponds to the standard states of an ideal gas at 1 bar and an ideal 1 M solution.

Density Functional Theory (DFT) calculations were performed using Turbomole software (versions 7.5.1,^45^ 7.7 ^46^ and 7.9 ^47^), and the DLPNO–CCSD­(T) calculations were performed using Orca software^48^ (v5.0.4 and 6.0). Conformer search was performed either manually or using COSMOconf^49^ software. The COSMO-RS^50,51^ approach as implemented in the COSMOtherm^52^ software (with BP_TZVPD_FINE_25 parametrization) was used for the calculation of solvation effects, taking into account both nonspecific and specific interactions. Further details are provided in the Supporting Information.

## Results and Discussion

### p*K*_a_ Values in 1,2-DFB from UV–vis Spectrophotometric Measurements

In this work, the p*K*_a_ values of 33 acids were determined, forming the first self-consistent acidity scale in 1,2-difluorobenzene. Each compound is linked to the scale with at least two independent Δp*K*_a_ measurements. Altogether, 64 Δp*K*_a_ measurements yielded a p*K*_a_ scale of acids spanning approximately 15.6 p*K*_a_ units. The acidity scale contains, among others, phenylmalononitriles, phenols, sulfonimides, and tetracyanopentadienes, meaning that CH-acids, OH-acids, as well as NH-acids are all present (Figure 2). The p*K*_a_ value of picric acid was initially arbitrarily set to zero to obtain the p*K*_a,rel_ values for all studied acids. The same was done by Paenurk et al. to obtain p*K*_a,rel_ values in 1,2-DCE.^21^ Another example of a similar approach is MeCN, where the absolute p*K*_a_ value of picric acid (11.00) is used as the anchor point of the p*K*_a_ scale.^19^

**Figure 2:**
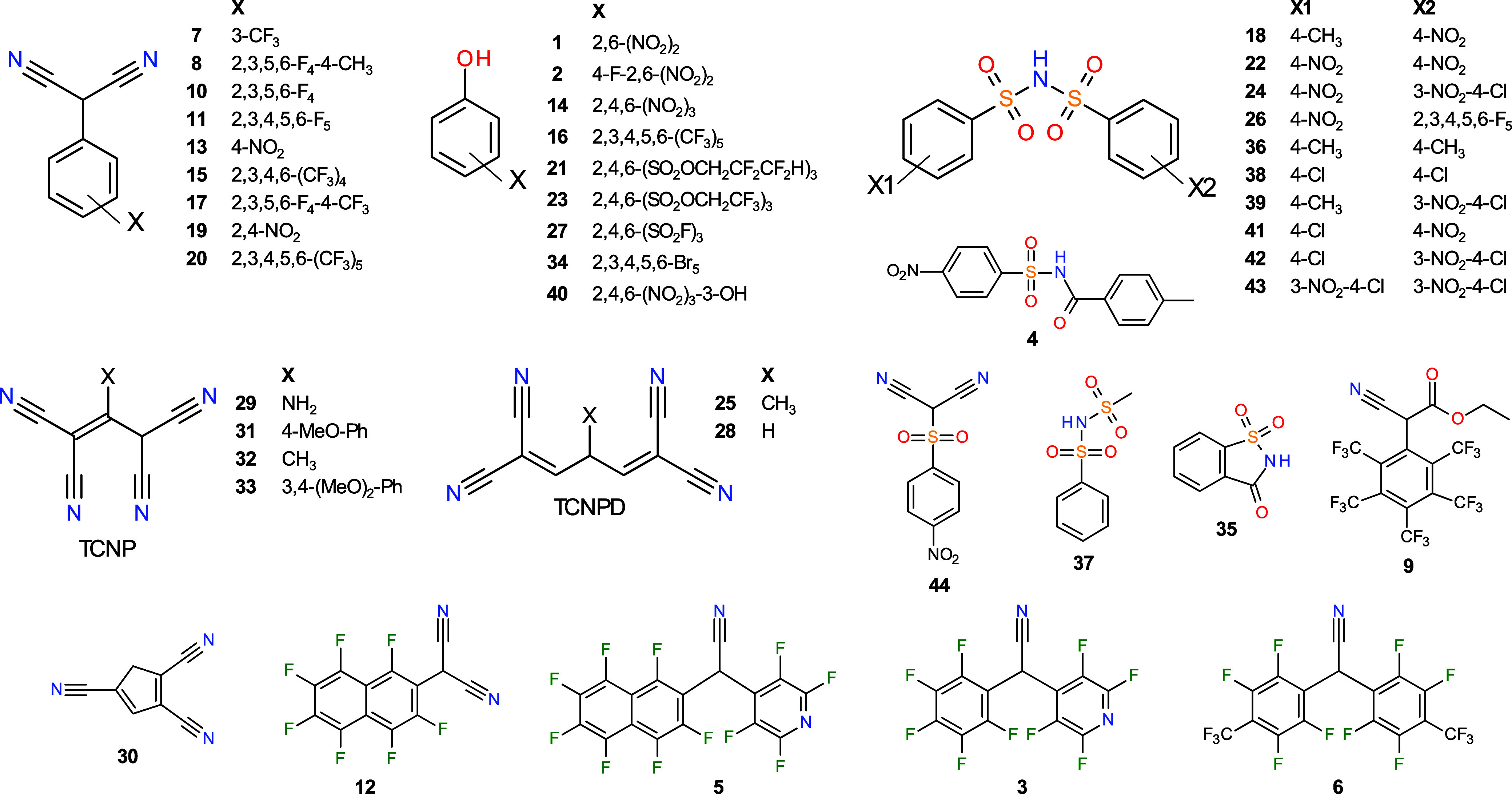
Structures of acids studied in this work.

To convert the relative p*K*_a,rel_ values to the absolute p*K*_a,DFB_ values (p*K*_a_ column of Table 1), computational p*K*_a_ values (p*K*_a,calc_ column of Table 1) of nine acids in 1,2-DFB, ranging from 30.6 to 49.3 were used as anchor points in this study. Least square minimization yielded an offset of 40.8 p*K*_a_ units, which was applied to reconstruct the scale in 1,2-DFB. The resulting absolute p*K*_a_ values now range from 32.0 to 47.6. The consistency standard deviation of the acidity scale in 1,2-DFB is 0.07 p*K*_a_ units. Although this consistency is higher than the consistencies of the acidity scales in 1,2-DCE and MeCN, where the *s* values are 0.04 and 0.03, respectively, it can still be considered adequate for an apolar solvent.^21,53^

There are two ways to express the uncertainty of the obtained p*K*_a_ values: “uncertainty relative to the scale” and “uncertainty of the absolute p*K*_a_ value”. The consistency standard deviation is a good proxy for the “uncertainty relative to the scale”. It can be considered as combined standard uncertainty estimate of the p*K*_a_ values for within-scale comparisons. It does not account for the large uncertainties of the computational values that were used for anchoring the scale to obtain the absolute p*K*_a_ value estimates—the contributions that “uncertainty of the absolute p*K*_a_ value” has to cover. Those uncertainties come from the large uncertainty of the Δ_solv_ *G*° of the proton in 1,2-DFB, as well as the computed Gibbs energies of the anions and neutrals of the acids. We expect the latter to be in the range of ca. 8 kJ mol^–1^ according to the study of Kröger et al.,^54^ but to at least partially cancel out in the p*K*_a_ calculation. The standard uncertainty of rCCC model by which Δ_solv_ *G*°(H^+^) value was obtained was estimated as 10 kJ mol^–1^ (1.8 p*K*_a_ units),^43,44^ but this estimate is based on significantly more polar solvents and may not apply to 1,2-DFB and other low-polarity solvents. At this moment, we are unable to provide a numerical uncertainty estimate for the absolute p*K*_a_ values, as there are no experimental Δ_solv_ *G*°(H^+^) values in very low-polarity media to reliably validate the computational methods, but it is certainly at least 2 p*K*_a_ units, and probably higher.

The p*K*_a_ value of 9.34 for acid **13** in 1,2-DFB has been previously reported by Kong et al.^10^ It is over 30 units lower than the p*K*_a_ of **13** presented in this work, and also by more than two units lower than its p*K*_a_ in MeCN (11.61 ^19^). It is highly unlikely (in fact all but impossible) that an acid has p*K*_a_ value in 1,2-DFB lower than in MeCN, because MeCN has a much higher polarity and ability to solvate the H^+^ ion, as well as the anions. The root cause for this large discrepancy in the paper by Kong et al. is that the absolute p*K*_a_ of their anchor compound (4-CF_3_SO_2_-C_6_H_4_CH­(CN)_2_) was determined as 8.13 ^10^ in 1,2-DFB. An absolute p*K*_a_ value in this range could be expected in a more polar solvent, e.g. MeCN, but is all but impossible in 1,2-DFB. These low values for **13** and 4-CF_3_SO_2_-C_6_H_4_CH­(CN)_2_ are almost certainly caused by basic impurities in the solvent or in the measured compounds (e.g., partial contamination of the acid by its salt), as well as by possible unrecognized flaws in the absolute p*K*_a_ measurement method by Kong et al.

Nevertheless, the difference in p*K*_a_ values of **13** and 4-CF_3_SO_2_-C_6_H_4_CH­(CN)_2_ of 1.21 units in their paper is fully realistic. Given these considerations, it is likely that on a relative scale, the p*K*_a_ values of the extensive set of acids reported by Kong et al. are appropriate. To make these literature p*K*_a_ values comparable with those in the current paper and thereby useful for practitioners, they were corrected by +31.7, based on the difference in p*K*_a_ values of **13** between this work (41.0) and Kong et al. (9.34). The corrected p*K*_a_ values are presented in Table 2. Our recommended p*K*_a_ values for 9-CN-fluorene (51.6) and 9-COOMe-fluorene (53.9) from Table 5, estimated by using the experimental 1,2-DFB values from this work, are in good agreement with the corrected literature values from Table 2 for these compounds, with 51.8 and 53.6, respectively. This strongly supports the claim that the experimentally determined relative acidities reported by Kong et al. and in this work agree when anchored to the same reference values.

**Table 2:** Corrected p*K*_a_ Values from Kong et al.^10^ in 1,2-DFB

acid^a^	p*K*_a_ values in 1,2-DFB from Kong et al.	
	original^b^	corrected^c^
2-(C_6_H_5_-C≡C-)-C_6_H_4_-COMe	27.68	59.4
C_6_H_5_-CO-CH_2_-C_6_H_5_	27.47	59.2
2-(C_6_H_5_-C≡C-)-C_6_H_4_-COEt	27.39	59.1
C_6_H_5_-CO-CH(Et)C_6_H_5_	27.32	59.0
C_6_H_5_-CO-CH(Me)C_6_H_5_	26.33	58.0
2-indanone	26.07	57.8
cyclopentadiene	26.00	57.7
MeCO-CH_2_-COMe	25.18	56.9
9-C_6_H_5_S-fluorene	25.14	56.8
4-MeO-C_6_H_4_CH_2_NO_2_	24.34	56.0
MeCO-CH_2_-COOEt	24.26	56.0
4-CN-C_6_H_4_CH_2_CN	24.32	56.0
MeCO-CH_2_-COOMe	24.14	55.8
9-(C_6_H_5_-NH-*N*=)-fluorene	23.94	55.6
(EtSO_2_)_2_(Et)CH	23.90	55.6
9-MeSO_2_-fluorene	23.49	55.2
(EtSO_2_)_2_(i-Pr)CH	23.31	55.0
9-EtSO_2_-fluorene	22.92	54.6
(EtSO_2_)_2_CH_2_	22.94	54.6
9-C_6_H_5_SO_2_-fluorene	22.85	54.5
[DBUH]^+^	22.58	54.3
9-COOMe-fluorene	21.93	53.6
(C_6_H_5_SO_2_)_2_CH_2_	21.85	53.5
C_6_H_5_-CO-CH_2_CN	20.95	52.6
4-Cl-C_6_H_4_-CO-CH_2_CN	20.10	51.8
9-CN-fluorene	20.15	51.8
2-NO_2_-9-Tos-fluorene	19.35	51.0
2-NO_2_-9-(4-Br-C_6_H_4_SO_2_)-fluorene	18.57	50.3
4-Me-2,6-(NO_2_)_2_-phenol	17.78	49.5
2-NO_2_-9-(2,4-Cl_2_-C_6_H_3_SO_2_)-fluorene	17.82	49.5
4-MeO-C_6_H_4_CH(CN)_2_	17.49	49.2
4-Me-C_6_H_4_CH(CN)_2_	17.10	48.8
9-CF_3_SO_2_-fluorene	16.76	48.5
C_6_H_5_CH(CN)_2_	16.36	48.1
4-Cl-2,6-(NO_2_)_2_-phenol	14.88	46.6
4-Cl-C_6_H_4_CH(CN)_2_	14.74	46.4
4-CF_3_-C_6_H_4_CH(CN)_2_	12.00	43.7
4-CN-C_6_H_4_CH(CN)_2_	10.23	41.9
4-NO_2_-C_6_H_4_CH(CN)_2_	9.34	41.0
4-CF_3_SO_2_-C_6_H_4_CH(CN)_2_	8.13	39.8

a Structures of the compounds can be found in ref 10. Tos represents the 4-Me-C_6_H_4_SO_2_–group.

b p*K*_a_ values as published by Kong et al.^10^

c All p*K*_a_ values were corrected by +31.7, the difference of p*K*_a_ values of **13** in this work (41.0) and Kong et al. (9.34).

Because many of the acids investigated in this work have been previously used to build p*K*_a_ scales in 1,2-DCE^21,33^ and MeCN^19^ (Table 3), correlating the results between these solvents and 1,2-DFB was possible (Table 4). p*K*_a_ values of the studied acids in 1,2-DFB correlate very well with p*K*_a_ values in both MeCN and 1,2-DCE. In Figure 3, the overall correlation between p*K*_a_ in 1,2-DFB and MeCN has a slope of 1.10 (*s* = 0.02). This shows the somewhat better differentiating ability of 1,2-DFB, which is expected because of the lower acidic and basic properties of 1,2-DFB compared to MeCN. A correlation using only phenylmalononitriles with a slope of 1.19 (*s* = 0.03) shows an even better differentiating ability of 1,2-DCE than MeCN. No clear grouping of data points representing different compound classes was observed with acids. The correlation between p*K*_a_ values in 1,2-DFB and 1,2-DCE, as shown in Figure 4, has a slope of 0.98 (*s* = 0.01), which means that the differentiating abilities of these solvents are almost indistinguishable. This is expected because of the much more similar polarity and lower acidic and basic properties of 1,2-DFB and 1,2-DCE compared to MeCN. Because of an insufficient amount of acidity data in 1,2-DFB, it was not possible to make compound class-specific correlations between 1,2-DCE and 1,2-DFB.

**Table 3:** Acidity Data of the Acids Examined in 1,2-DFB, Together with Literature Values in MeCN and 1,2-DCE

no	acid^a^	p*K*_a_ values in		
		1,2-DFB	MeCN^19^	1,2-DCE^21^
**1**	2,6-(NO_2_)_2_-phenol	47.6	16.74	
2	4-F-2,6-(NO_2_)_2_-phenol	46.7	16.17	
3	(4-NC_5_F_4_)(C_6_F_5_)CHCN	46.1	16.39	50.5
4	4-NO_2_-C_6_H_4_SO_2_NHCOC_6_H_4_-4-Me	45.6	15.68	
5	(4-NC_5_F_4_)(2-C_10_F_7_)CHCN	45.6	16.01	
6	(4-CF_3_-C_6_F_4_)_2_CHCN	45.6	16.12	
7	3-CF_3_-C_6_H_4_CH(CN)_2_	44.6	14.70	49.0
8	4-Me-C_6_F_4_CH(CN)_2_	44.1	13.87	
9	2,3,4,5,6-(CF_3_)_5_-C_6_CH(CN)COOEt	43.4	13.41	47.8
10	4-H-C_6_F_4_CH(CN)_2_	43.0	12.98	47.5
11	C_6_F_5_CH(CN)_2_	42.8	13.01	
12	2-C_10_F_7_CH(CN)_2_	41.9	12.23	46.3
13	4-NO_2_-C_6_H_4_CH(CN)_2_	41.0	11.61	
14	picric acid	40.8	11.00	45.0
15	2,3,4,6-(CF_3_)_4_-C_6_HCH(CN)_2_	39.9	10.42	44.2
16	2,3,4,5,6-(CF_3_)_5_-phenol	39.9	10.46	
17	4-CF_3_-C_6_F_4_CH(CN)_2_	39.7	10.19	44.2
18	4-NO_2_-C_6_H_4_SO_2_NHTos	39.4	10.04	43.3
19	2,4-(NO_2_)_2_-C_6_H_3_CH(CN)_2_	38.5	9.58	
20	2,3,4,5,6-(CF_3_)_5_-C_6_CH(CN)_2_	37.8	8.88	42.2
21	2,4,6-(SO_2_OCH_2_CF_2_CF_2_H)_3_-phenol	36.9	8.14	
22	(4-NO_2_-C_6_H_4_-SO_2_)_2_NH	36.8	8.15	41.1
23	2,4,6-(SO_2_OCH_2_CF_3_)_3_-phenol	36.6	7.94	
24	4-NO_2_-C_6_H_4_SO_2_NHSO_2_C_6_H_3_-3-NO_2_-4-Cl	36.5	7.82	40.7
25	Me-TCNPD	36.3	7.36	
26	C_6_F_5_SO_2_NHSO_2_C_6_H_4_-4-NO_2_	35.4	6.60	
27	2,4,6-(SO_2_F)_3_-phenol	34.7	5.22	39.0
28	H-TCNPD	34.7	5.64	
29	NH_2_-TCNP	33.9		38.2
30	2,3,5-(CN)_3_-cyclopentadiene	33.6	3.65	38.0
31	4-MeO-C_6_H_4_-TCNP	32.0		36.1
32	Me-TCNP	32.1		36.4
33	3,4-(MeO)_2_-C_6_H_3_-TCNP	32.0		36.1

a Tos represents the 4-Me-C_6_H_4_SO_2_–group; TCNPD - 1,1,5,5-tetracyanopentadiene; TCNP −1,1,3,3-tetracyanopropene.

**Table 4:** Equations for Correlations of p*K*_a_ Values of Acids between 1,2-DFB and Other Solvents

solvent	N	regression equation	σ	*r* ^2^
acetonitrile (MeCN)				
all compounds	29	p*K*_a, DFB_ = 1.10(0.02) p*K*_a, MeCN_ + 28.41(0.26)	0.44	0.9891
phenylmalononitriles	10	p*K*_a, DFB_ = 1.19(0.03) p*K*_a, MeCN_ + 27.31(0.38)	0.19	0.9943
1,2-dichloroethane (DCE)				
all compounds	18	p*K*_a, DFB_ = 0.98(0.01) p*K*_a, DCE_ – 3.64(0.34)	0.15	0.9990

**Figure 3:**
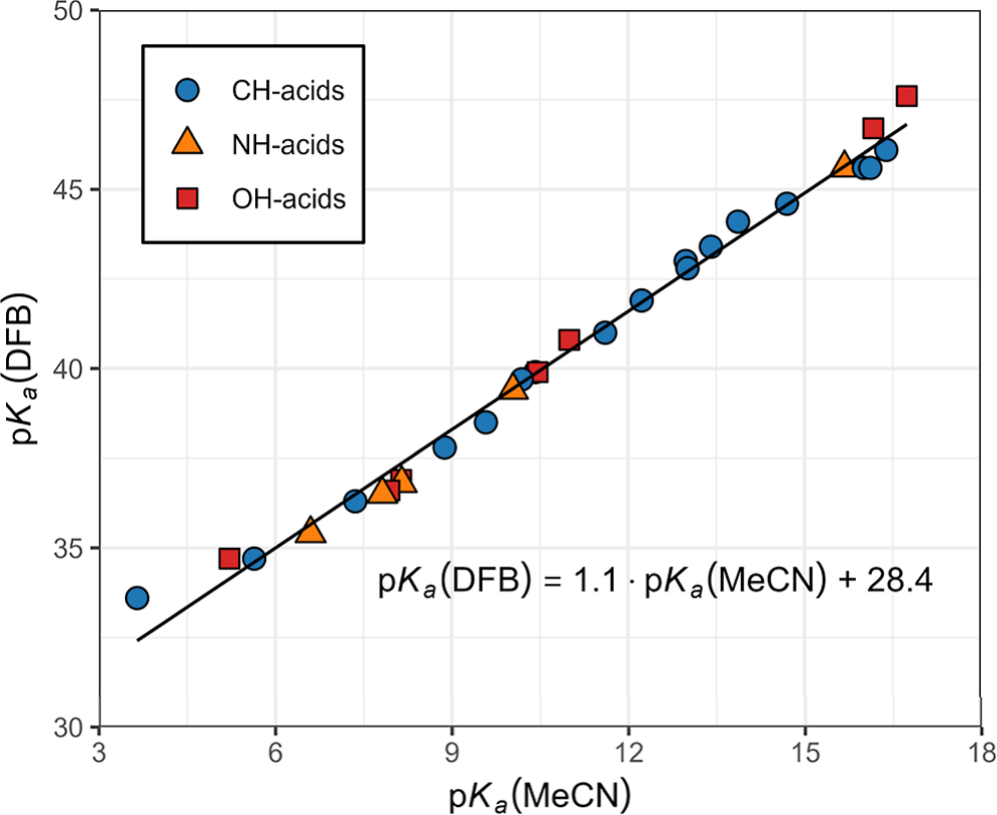
Correlation of p*K*_a_ values of acids in 1,2-DFB and acetonitrile.

**Figure 4:**
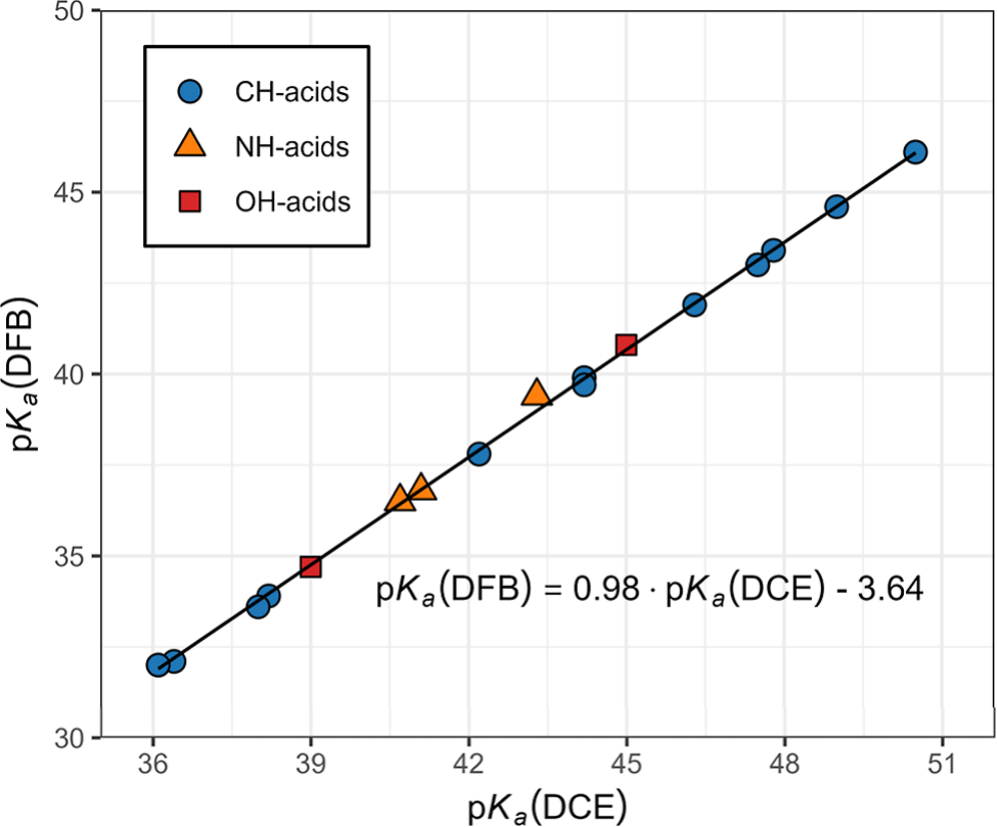
Correlation of p*K*_a_ values of acids in 1,2-DFB and 1,2-DCE.

The good correlations between p*K*_a_ values in 1,2-DFB and those in MeCN and 1,2-DCE allow for the estimation of p*K*_a_ values in 1,2-DFB for compounds not directly measured. p*K*_a_ values in 1,2-DFB have been estimated for 104 additional compounds (Table 5). Among these compounds, p*K*_a_ values are available for 36 compounds in both MeCN and 1,2-DCE, while 36 compounds have values only in MeCN and 32 only in 1,2-DCE. The estimated p*K*_a_ values of these compounds range from 25.6 to 54.3. The compounds belong to several classes, including substituted phenols, malononitriles, sulfonimides, TCNPs, binary acids, and oxoacids. For compounds with p*K*_a_ values available in both MeCN and 1,2-DCE, as shown in Table 5, the recommended p*K*_a_ values in 1,2-DFB were calculated as the average of the two values, while for compounds with only a single available value, that value was taken as the recommended p*K*_a_.

**Table 5:** p*K*_a_ Values in 1,2-DFB Estimated from p*K*_a_ in Other Solvents

compounds^a^	p*K*_a_ values in 1,2-DFB estimated		recommended p*K*_a_ values in 1,2-DFB	assigned standard uncertainty^b^
	from MeCN p*K*_a_ values	from 1,2-DCE p*K*_a_ values		
9-COOMe-fluorene	54.3	53.6	53.9	0.5
(4-Me-C_6_F_4_)_2_CHCN	53.5	53.2	53.3	0.5
(4-Me-C_6_F_4_)(C_6_F_5_)CHCN	52.5	52.2	52.4	0.5
9-CN-fluorene	51.9	51.4	51.6	0.5
(4-H-C_6_F_4_)(C_6_F_5_)CHCN	51.6	51.2	51.4	0.5
(4-Cl-C_6_F_4_)(C_6_F_5_)CHCN	50.8	50.3	50.5	0.5
2,3,4,5,6-F_5_-phenol	50.5		50.5	1.0
(2-C_10_F_7_)(C_6_F_5_)CHCN	50.5	49.9	50.2	0.5
(2-C_10_F_7_)_2_CHCN	49.6	48.9	49.3	0.5
9-C_6_F_5_-octafluorofluorene	49.2	49.3	49.2	0.5
(4-NC_5_F_4_)(4-CF_3_-C_6_F_4_)NH	48.5		48.5	1.0
(4-CF_3_-C_6_F_4_)(C_6_F_5_)CHCN	48.3	47.9	48.1	0.5
4-C_6_F_5_-2,3,5,6-F_4_-phenol	48.3		48.3	1.0
2,3,4,5,6-Cl_5_-phenol	48.2		48.2	1.0
2,3,4,5,6-Br_5_-phenol	48.0		48.0	1.0
(C_6_F_5_)CH(CN)COOEt	47.9	48.0	48.0	0.5
4-Me-C_6_H_4_-CH(CN)_2_	47.7		47.7	1.0
(2-C_10_F_7_)CH(CN)COOEt	47.6	47.6	47.6	0.5
(4-Cl-C_6_F_4_)CH(CN)COOEt	47.5	47.8	47.6	0.5
(4-NC_5_F_4_)_2_NH	47.3		47.3	1.0
2-Me-4,6-(NO_2_)_2_-phenol	47.1		47.1	1.0
2,4-(NO_2_)_2_-phenol	46.7		46.7	1.0
4-CF_3_-2,3,5,6-F_4_-phenol	46.7		46.7	1.0
(4-CF_3_-C_6_F_4_)CH(CN)COOEt	46.1	46.2	46.1	0.5
(4-NC_5_F_4_)CH(CN)COOEt	44.8	45.0	44.9	0.5
saccharin	44.4		44.4	1.0
3-NO_2_-C_6_H_4_SO_2_NHSO_2_C_6_H_4_-3-Cl	44.4		44.4	1.0
4-NO_2_-C_6_H_4_SO_2_NHSO_2_C_6_H_4_-3-Cl	44.3		44.3	1.0
2-Cl-4,6-(NO_2_)_2_-phenol	44.1		44.1	1.0
(4-NC_5_F_4_)_2_CHCN	43.2	43.0	43.1	0.5
Tos_2_NH	41.6		41.6	1.0
Ph-SO_2_NHSO_2_-Me	41.5		41.5	1.0
bromothymol blue	41.3	41.7	41.5	0.5
(C_6_H_5_SO_2_)_2_NH	40.9		40.9	1.0
4-MeO-C_6_H_4_SO_2_NHSO_2_C_6_H_4_-4-Cl	40.8		40.8	1.0
4-Cl-C_6_H_4_SO_2_NH-Tos	40.6		40.6	1.0
bromocresol green	40.5	40.9	40.7	0.5
HNO_3_	40.1	39.3	39.7	0.5
(2,4,6-(NO_2_)_3_-C_6_H_2_)_2_NH	40.0		40.0	1.0
HCl	39.7	40.8	40.3	0.6
4-Cl-C_6_H_4_SO_2_NH-Tos	39.7		39.7	1.0
(4-Cl-C_6_H_4_-SO_2_)_2_NH	39.6		39.6	1.0
4-Cl-3-NO_2_C_6_H_3_SO_2_NH-Tos	39.1		39.1	1.0
4-NO_2_-C_6_H_4_SO_2_NHSO_2_C_6_H_4_-4-Cl	38.5	38.1	38.3	0.5
(2,3,4,5,6-(CF_3_)_5_-C_6_)_2_NH	38.0		38.0	1.0
4-Cl-3-NO_2_-C_6_H_3_SO_2_NHSO_2_C_6_H_4_-4-Cl	38.0		38.0	1.0
TosOH	37.8		37.8	1.0
3-NO_2_-C_6_H_4_SO_2_NHSO_2_C_6_H_3_-3-NO_2_-4-Cl	37.0		37.0	1.0
H_2_SO_4_	36.8	38.4	37.6	0.9
C_6_F_5_SO_2_NHSO_2_C_6_H_4_-4-Cl	36.7		36.7	1.0
(3-NO_2_-4-Cl-C_6_H_3_SO_2_)_2_NH	36.6	36.1	36.4	0.5
C_6_F_5_SO_2_NHSO_2_C_6_H_3_-3-NO_2_-4-Cl	35.2		35.2	1.0
4-NO_2_-C_6_H_4_SO_2_CH(CN)_2_	35.0	35.6	35.3	0.5
Tos-NH-Tf	34.8		34.8	1.0
4-Me-C_6_H_4_-C(=NTf)-NH-Tf	34.8		34.8	1.0
C_6_H_5_-C(=NTf)-NH-Tf	34.7		34.7	1.0
C_6_H_5_SO_2_NH-Tf	34.5		34.5	1.0
HBr	34.3	36.3	35.3	1.1
4-F-C_6_H_4_-C(=NTf)-NH-Tf	34.2		34.2	1.0
4-Cl-C_6_H_4_-C(=NTf)-NH-Tf	34.1		34.1	1.0
(C_6_F_5_SO_2_)_2_NH	34.0		34.0	1.0
CH(CN)_3_	33.9	34.4	34.2	0.5
4-Cl-C_6_H_4_SO(=NTf)-NH-Tos	33.7	33.8	33.7	0.5
2,4,6-Tf_3_-phenol	33.3	34.2	33.7	0.5
C(CN)_2_=C(CN)OH	33.2	32.1	32.6	0.7
4-NO_2_-C_6_H_4_SO_2_NHTf	32.9	32.9	32.9	0.5
4-Cl-C_6_H_4_SO(=NTf)NHSO_2_C_6_H_4_-4-Cl	32.8	32.9	32.9	0.5
2,4-(NO_2_)_2_-C_6_H_3_SO_2_OH	32.8	31.8	32.3	0.6
4-Cl-C_6_H_4_SO(=NTf)NHSO_2_C_6_H_4_-4-NO_2_	32.0	31.7	31.9	0.5
HI	31.5	33.5	32.5	1.1
TfOH	31.3	29.6	30.4	0.9
HClO_4_	30.1	28.1	29.1	1.1
styphnic acid		39.8	39.8	1.0
pentacyanophenol		33.2	33.2	1.0
C_6_F_5_CH(Tf)_2_		31.7	31.7	1.0
HB(CN)(CF_3_)_3_		31.5	31.5	1.0
Ph-TCNP		31.3	31.3	1.0
HBF_4_		30.8	30.8	1.0
FSO_2_OH		30.6	30.6	1.0
3-CF_3_-C_6_H_4_-TCNP		30.2	30.2	1.0
[C_6_H_5_SO(=NTf)]_2_NH		29.5	29.5	1.0
[(C_2_F_5_)_2_PO]_2_NH		29.3	29.3	1.0
2,4,6-(NO_2_)_3_-C_6_H_2_SO_2_OH		29.4	29.4	1.0
[C(CN)_2_=C(CN)]_2_CH_2_		29.4	29.4	1.0
C_6_H_5_SO(=NTf)NHTf		29.2	29.2	1.0
TfCH(CN)_2_		29.3	29.3	1.0
Br-TCNP		29.1	29.1	1.0
[C(CN)_2_=C(CN)]_2_NH		28.9	28.9	1.0
3,5-(CF_3_)_5_-C_6_H_3_-TCNP		28.8	28.8	1.0
Tf_2_NH		28.9	28.9	1.0
4-Cl-C_6_H_4_SO(=NTf)NHTf		28.6	28.6	1.0
Cl-TCNP		28.8	28.8	1.0
(C_3_F_7_SO_2_)_2_NH		28.6	28.6	1.0
(C_4_F_9_SO_2_)_2_NH		28.5	28.5	1.0
CN-CH_2_-TCNP		28.5	28.5	1.0
(C_2_F_5_SO_2_)_2_NH		28.4	28.4	1.0
CF_3_-TCNP		28.1	28.1	1.0
CF_2_(CF_2_SO_2_)_2_NH		27.8	27.8	1.0
4-NO_2_-C_6_H_4_SO(=NTf)NHTf		27.5	27.5	1.0
HB(CN)_4_		27.6	27.6	1.0
(FSO_2_)_3_CH		27.3	27.3	1.0
Tf_2_CH(CN)		25.9	25.9	1.0
2,3,4,5-tetracyanocyclopentadiene		25.8	25.8	1.0
CN-TCNP		25.6	25.6	1.0

a Tos represents the 4-Me-C_6_H_4_SO_2_– group; Tf represents the CF_3_SO_2_- group; TCNP −1,1,3,3-tetracyanopropene.

b All these uncertainties have to be interpreted as “uncertainties relative to the scale”.

The standard uncertainties were also assigned to the estimated p*K*_a_ values. For compounds with p*K*_a_ values measured in both MeCN and 1,2-DCE, the individual uncertainty was calculated as half the absolute difference between the two values, with an additional 0.1 p*K*_a_ unit added to account for systematic effects. The root-mean-square (RMS) of these individual uncertainties, 0.5 p*K*_a_ units, was used as a baseline. If a compound’s individual uncertainty was smaller than this baseline, the baseline uncertainty value was applied; otherwise, the calculated individual uncertainty was used. Furthermore, for compounds with only a single estimated p*K*_a_ value from either MeCN or 1,2-DCE, an uncertainty of 1.0 p*K*_a_ unit was assigned, which provides a reasonable and conservative uncertainty estimate for the recommended p*K*_a_ values in 1,2-DFB. All these uncertainties have to be interpreted as “uncertainties relative to the scale”.

### Acidity Scale in 1,2-DFB Using Differential Potentiometry

A total of 75 potentiometric measurements, shown as black double-headed arrows in Table 6, were used to create a pH ladder in 1,2-DFB. The black arrows indicate measurement series that meet the above-described quality criteria and are consistent with other measurement series. The red arrows indicate measurements that met the quality criteria but were strongly inconsistent (by 0.75 pH unit) with the remaining measurements and contributed significantly to a large deviation in the ladder’s overall consistency standard deviation. As a result, these measurements were removed from the least-squares minimization process to assign pH_abs_ values to each solution. However, we considered it important to show such measurements on the ladder.

**Table 6:**
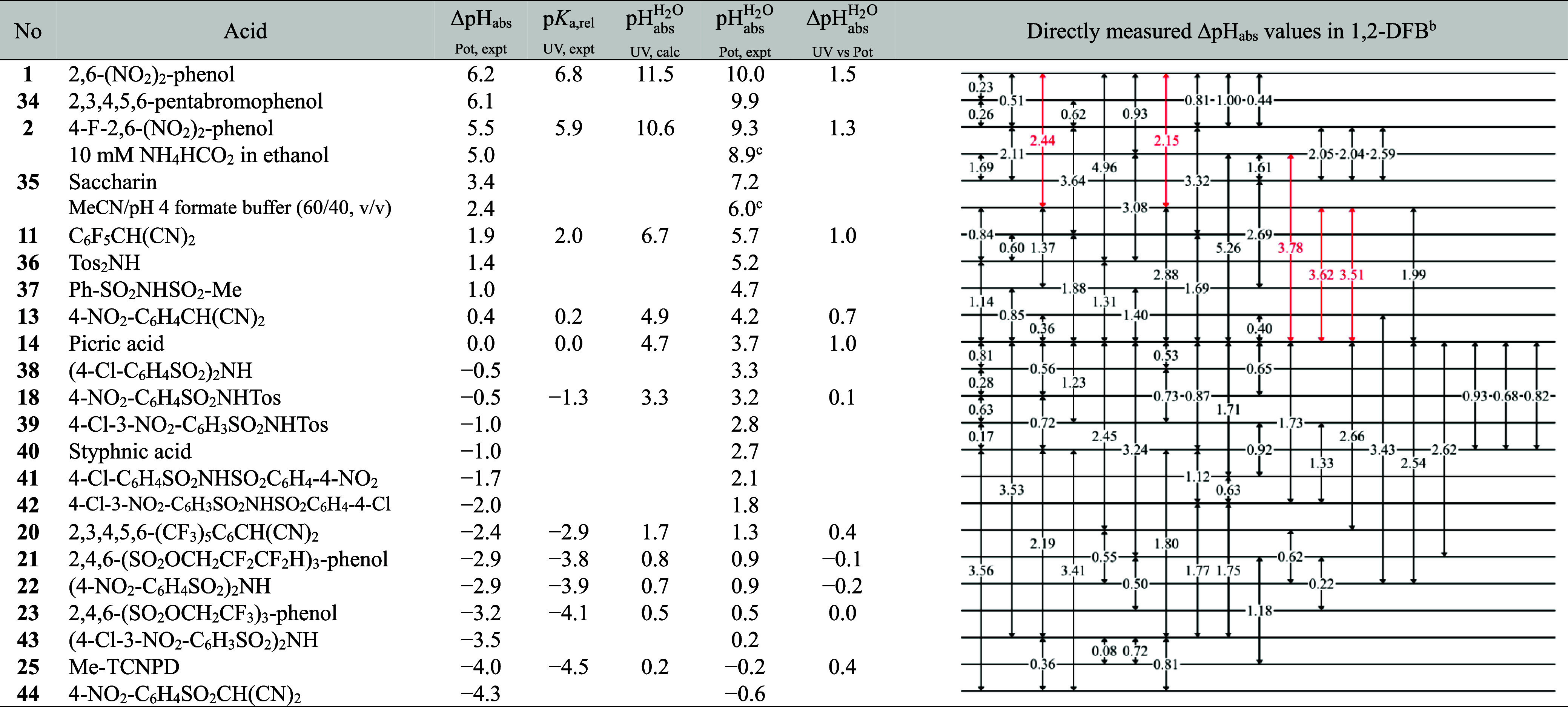
pH_abs_ Scale of Solutions in 1,2-DFB Obtained Using Differential Potentiometry^a^

a ΔpH_abs_ (Pot, expt) are the pH_abs_ differences vs picric acid, obtained from the directly measured ΔpH_abs_ values using the least-squares procedure; p*K*_a,rel_ (UV, expt) are the p*K*_a,rel_ values (vs picric acid) of the compounds that were used to find the ${pH}_{abs}^{H_{2}O}$ (UV, calc) values; ${pH}_{abs}^{H_{2}O}$ (UV, calc) are the ${pH}_{abs}^{H_{2}O}$ found by combining the experimental p*K*_a,rel_ values and computational p*K*_a_ values of the compounds; ${pH}_{abs}^{H_{2}O}$ (Pot, expt) are the experimental ${pH}_{abs}^{H_{2}O}$ values from potentiometric ΔpH_abs_ measurements, anchored to the ${pH}_{abs}^{H_{2}O}$ values of 10 mM NH_4_HCO_2_ in ethanol and mixture of MeCN and aqueous pH 4 formate buffer (60/40) from literature; Δ ${pH}_{abs}^{H_{2}O}$ (UV vs Pot) are the differences between the ${pH}_{abs}^{H_{2}O}$ (UV, calc) and ${pH}_{abs}^{H_{2}O}$ (Pot, expt) values.

b The acids were used as equimolar buffer solutions of the acid and salt at 0.333 mM concentration. The black arrows were used for the pH_abs_ assignment, while the red arrows were left out of the least-squares minimization due to systematic deviation.

c The solutions, which ${pH}_{abs}^{H_{2}O}$ values were used as anchor points for obtaining the ${pH}_{abs}^{H_{2}O}$ (Pot, expt) values.

Not all compounds included in this study were studied using differential potentiometry due to two main factors. First, most compounds were available in limited quantities (sometimes just few milligrams), and preparing solutions for potentiometric measurements requires ten times higher concentrations and thus ten times more compound compared to the spectrophotometric method. Second, preparing solutions with the required high concentration is often hindered by the limited solubility of many compounds in 1,2-DFB, particularly for compounds in salt form. These compounds either do not dissolve completely or form turbid solutions when bistriflimide is added. Most compounds in the ladder also needed a long time (24–48 h) to completely dissolve in 1,2-DFB at a suitable concentration.

The overall consistency standard deviation of the pH_abs_ scale in 1,2-DFB is 0.30 pH units. This value can be considered a suitable measurement uncertainty estimate for comparing ${pH}_{abs}^{H_{2}O}$ values within this 1,2-DFB ${pH}_{abs}^{H_{2}O}$ scale.

It is higher compared to the previous study in another nonpolar solvent, 1,2-dichloroethane, where the consistency standard deviation was 0.17 pH units.^20^ Meanwhile, the pH_abs_ measurements of chromatographic mobile phases (mixtures of methanol, acetonitrile, and water) reported consistency standard deviations of 0.14 ^55^ and 0.09 ^56^ pH units. The large consistency standard deviation in this work likely reflects the difficulty of obtaining stable and reproducible potential readings in 1,2-DFB.

The pH_abs_ scale in 1,2-DFB consists of 22 compounds that span from −0.6 to 10.0. The assigned pH_abs_ values have been anchored to the previously determined ${pH}_{abs}^{H_{2}O}$ values of two “bridging” solutions and are thus comparable to the conventional aqueous pH scale and are denoted as ${pH}_{abs}^{H_{2}O}$ values in Table 6. One limitation of performing differential potentiometric measurements in low-polarity media such as 1,2-DFB is its exposure to water. Direct comparison against aqueous solutions is impossible, as very minute contamination of water in 1,2-DFB solutions could drastically alter its pH. To solve this, two bridging solutions with known ${pH}_{abs}^{H_{2}O}$ values were measured against 1,2-DFB solutions: an acetonitrile/pH 4.0 formate (60/40 v/v) solution and a 10 mM ammonium formate solution in absolute ethanol. Their known ${pH}_{abs}^{H_{2}O}$ values were 6.0 ^56^ and 8.9,^57^ respectively, which are also obtained through the differential potentiometry method, measured against aqueous standard buffer solutions.

The uncertainty of the obtained ${pH}_{abs}^{H_{2}O}$ values for comparing with ${pH}_{abs}^{H_{2}O}$ values in other solvents can be estimated in simplified terms as consisting of three components: (1) standard uncertainties of the ${pH}_{abs}^{H_{2}O}$ values of the bridging solutions, which can be estimated as 0.13 pH units;^56^ (2) the average standard uncertainty due to the liquid junction potential cancellation assumption estimated as 6.3 mV,^58^ corresponding to 0.11 pH units and (3) the consistency standard deviation of the scale of 0.30 pH units. The combined standard uncertainty estimate is computed as root sum square of these contributions and is 0.34 pH units. This is a crude uncertainty estimate. On one hand, at least part of the consistency standard deviation is likely caused by incomplete cancellation of the liquid junction potential, leading to some double counting and overestimation of uncertainty. On the other hand, the uncertainty due to the liquid junction potential cancellation assumption has been estimated^58^ based on the measurements in different solvents that are all significantly more polar than 1,2-DFB. Thus, it is likely somewhat underestimated in the case of 1,2-DFB. Thus, we recommend rounding this uncertainty estimate upward to 0.4 pH units.

### Comparison of Potentiometric pH_abs_ Values and p*K*_a_-Derived pH_abs_ Values in 1,2-DFB

For comparison and verification purposes, the ${pH}_{abs}^{H_{2}O}$ values of equimolar buffer solutions were also computed from the p*K*_a_ values in 1,2-DFB obtained from UV–vis spectrophotometry and the solvation Gibbs energies of the hydrogen ion in water (−1105 kJ mol^–1^ with uncertainty of 8 kJ mol^–1^, uncertainty type or coverage probability not indicated)^59^ and in 1,2-DFB (−899 kJ mol^–1^ with an estimated standard uncertainty of 10 kJ mol^–1^ but in reality possibly higher, see above) .^43,44^ This approach enables direct comparison with ${pH}_{abs}^{H_{2}O}$ values obtained by differential potentiometry. As shown in Table 6, the p*K*_a_-derived ${pH}_{abs}^{H_{2}O}$ values are on an average higher, with the root-mean-square average difference of 0.8 pH units. Considering the large estimated uncertainties of the Δ_solv_ *G*° values of H^+^ in water and 1,2-DFB, corresponding to 1.4 and at least 1.8 pH units, respectively, as well as the above-described uncertainties inherent in both UV–vis spectrophotometric and potentiometric measurements, the agreement can be considered good. This agreement between ${pH}_{abs}^{H_{2}O}$ values from two completely independent routes (experimental potentiometry anchored to aqueous pH standards and quantum chemical computations together with UV–vis spectrophotometry) demonstrates that despite the high uncertainties involved, the underlying theoretical considerations are solid.

The discrepancy between the two sets of ${pH}_{abs}^{H_{2}O}$ values is more pronounced for compounds with smaller molecular size. This behavior can be attributed to the weak ion-solvating ability of 1,2-DFB, in which acids exhibit limited dissociation and instead form significant ion–ion interactions and aggregates.^21^ In addition, the higher acid concentrations employed in the potentiometric measurements compared to the UV–vis spectrophotometric method further enhance aggregation effects, contributing to the observed differences between the two approaches.^20^

## Conclusions

Experimental investigation of Brønsted acid–base equilibria is fully feasible in 1,2-difluorobenzene (1,2-DFB) with UV–vis spectrophotometry and differential potentiometry. 33 p*K*_a_ values have been directly measured in 1,2-DFB using spectrophotometry. These p*K*_a_ values correlate very well (*r* ^2^ values close to 0.99 or higher) with p*K*_a_ values in acetonitrile and 1,2-dichloroethane, enabling p*K*_a_ predictions. 104 additional p*K*_a_ values in 1,2-DFB were thus obtained in this work. The p*K*_a_ values 1,2-DFB were all between 25 and 54. Thus, spontaneous acid dissociation in 1,2-DFB does not occur. At the same time, with sufficiently strong bases, acids can be easily deprotonated.

An experimental pH_abs_ scale spanning more than 10 orders of magnitude has been established using differential potentiometry. When aligned with the aqueous pH scale, the resulting ${pH}_{abs}^{H_{2}O}$ values range from −0.6 to 10.0, meaning that strongly acidic media (in terms of the solvated proton activity) are possible in 1,2-DFB.

As an important result, good agreement was observed between the potentiometrically measured ${pH}_{abs}^{H_{2}O}$ values and ${pH}_{abs}^{H_{2}O}$ values calculated from the p*K*_a_ values. This agreement between two completely independent routes (experimental potentiometry anchored to aqueous pH standards and quantum chemical computations together with UV–vis spectrophotometry) demonstrates that, despite the high uncertainties involved, the underlying theoretical considerations are solid.

These results open the possibility of quantitatively describing and measuring Brønsted acid–base processes in 1,2-DFB, including potentiometric and UV–vis spectrophotometric ${pH}_{abs}^{H_{2}O}$ measurement.

## Supplementary Material



## Data Availability

The data underlying this study are openly available in Zenodo at 10.5281/zenodo.18216265 and its Supporting Information. p*K*_a_ measurement and minimization results; p*K*_a_ UV–vis spectra measurements as a set of .xlsx files; Solvent correlations and estimated p*K*_a_ values in 1,2-DFB; Computational energies in the gas phase, solvation energies and calculated p*K*_a_ values in 1,2-DFB; Optimized geometries as sets of .xyz, .cosmo and .energy files; Individual pH_abs_ potentiometric measurement results as a set of .csv files; pH_abs_ measurement and minimization results

## Notes

Detailed description of theoretical p*K*_a_ computation methodology and UV–vis spectra of all compounds used for p*K*_a_ measurements (PDF)
