# Supplementary material for: Acidity Scale in 1,2-Difluorobenzene
Source: ACS Omega. 2026 May 11;11(20):30073–86. doi: 10.1021/acsomega.6c02089 (PMC13216963; doi:10.1021/acsomega.6c02089)
Supplement: Supplementary file 1 [file ao6c02089_si_001.pdf]

SUPPORTING INFORMATION

Acidity Scale in 1,2-Difluorobenzene

John Paulo Samin,* Helerin Roomet, Märt Lõkov, Sofja Tshepelevitsh,

Jaan Saame, Agnes Heering, Ivo Leito*

Institute of Chemistry, University of Tartu, Ravila 14a, 50411 Tartu, Estonia

E-mail of the corresponding author: john.paulo.aquino.samin@ut.ee, ivo.leito@ut.ee

Table of Contents

I. Computational methodology S2

A. Software S2

B. Calculation of pK_a values S2

C. Calculation of ionic radii S3

II. References S4

III. UV-Vis spectra of all compounds used for pK_a measurements S5

I. Computational methodology

A. Software

DFT calculations were carried out using Turbomole software (V7.5.1[1], V7.7[2] and V7.9[3]). Conformer search was performed either manually or using COSMOconf[4] software. Orca[5] software (versions 5.0.4 and 6.0.0) was used for DLPNO-CCSD(T) calculations. COSMOtherm[6] software (with parametrization BP_TZVPD_FINE_25) was used for calculation of solvation effects.

B. Calculation of pK_a values

The computational pK_a values in 1,2-difluorobenzene were obtained by combining gas-phase free energies and solvation free energies[7] as follows:

$$AH \rightleftarrows A^- + H^+ \qquad (S1)$$

$$pK_a = -\log([A^-][H^+]/[AH]) = \Delta G_{diss} / RT\ln(10) \qquad (S2)$$

$$\Delta G_{diss} = [G_{gas}(A^-) + \Delta_{solv}G°(A^-)] + [G_{gas}(H^+) + \Delta_{solv}G°(H^+)] - [G_{gas}(AH) + \Delta_{solv}G°(AH)] \qquad (S3)$$

$$G_{gas} = E(CBS) + En + RT - TS \qquad (S4)$$

$$\Delta_{solv}G° = RT\ln(p \cdot x) \qquad (S5)$$

Where

AH – neutral acid;
A^- – anion;
H^+ – proton;
ΔG_{diss} – Gibbs energy change of acid dissociation reaction (eq. S1) in solution;
R – universal gas constant;
T – temperature;
G_{gas} – Gibbs energy of the particle in the gas phase; **$G_{gas}(H^+)$** = −26.3 kJ mol^{-1} [8]
$\Delta_{solv}G°$ – Gibbs energy of solvation; **$\Delta_{solv}G°(H^+)$** = −898.8 kJ mol^{-1} [9]
E(CBS) – complete basis set extrapolated electronic energy;
En – contributions to internal thermal energy in the gas phase;
S – entropy in the gas phase;
p – partial vapor pressure of compound in solution, computed at infinite dilution;
x – mole fraction of an ideal one-molar solution as the standard state.

Calculation of $\mathbf{G_{gas}}$ (eq. S4):

For each species, conformer search was performed to identify the most stable conformer in the gas phase. Geometries of neutral (AH) and anionic (A^-) species were optimised and vibrational frequency analysis carried out at (RI)BP86/def2-TZVPP level of theory with DFT-D3(BJ) dispersion correction (SCF convergence criterion: 10^{-6} H, grid: m3). The absence of imaginary frequencies in the vibrational spectra was taken as proof that the geometry corresponds to local energy minimum. Thermal energy contributions (**En**) and entropy (**S**) were computed using freeh module of Turbomole. DLPNO-CCSD(T)[10] single-point calculations were carried out with Dunning's basis sets cc-pVDZ, cc-pVTZ, and cc-pVQZ. The complete basis set energy values **E(CBS)** were found based on the procedure developed by Helgaker et al[11]: as an intercept of energy vs X^{-3} plot, where X = 2 for cc-pVDZ, X = 3 for cc-pVTZ and X = 4 for cc-pVQZ.

Calculation of $\mathbf{\Delta_{solv}G°}$ (eq. S5):

Conformer search was performed to identify important conformers (abundance over ~5% in solution). Geometry optimization and vibrational frequency analysis were carried out at (RI)BP86/def-TZVP level in the ideal conductor (COSMO model) and in the gas phase, followed by single-point energy calculations at (RI)BP86/def2-TZVPD level (with and without COSMO model, respectively). SCF convergence criterion of 10^{-6} H and grid m3 were used in most cases, changed to 10^{-7} H and grid m4 for re-optimization in a few cases where imaginary frequencies were found after initial optimization. Persistent imaginary frequency of −3 cm^{-1} was tolerated in case of one geometry in ideal conductor (anion of $(4\text{-}NO_2\text{-}C_6H_4SO_2)_2NH$). The obtained energies in conductor and gas phase, as well as partial charge distributions on molecular surface in ideal conductor, serve as input to statistical thermodynamic calculation by COSMO-RS method[12–14] that yield partial pressures over the mixture (**p**) values ("Log10(partial pressure [mbar])" in Cosmotherm output). All important conformers were used for these calculations (automatically weighting by COSMOtherm according to their energies in solution).

C. Calculation of ionic radii

Ionic radii were calculated using structures optimised at (RI)BP86/def-TZVP level of theory in ideal conductor (COSMO model). Comprehensive conformer search was not performed in all cases, rather, the conformer assumed to be prevalent/abundant in 1,2-difluorobenzene was used. Radii were calculated by three methods:

(1) from molecular volume using the sphere volume formula;

(2) from the dimensions of an imaginary cuboid inside which an ion's molecular surface would exactly fit (side lengths averaged and divided by two);

(3) as an average distance of surface segments (as listed in *.cosmo file) from their centroid.

The values obtained by the three methods were averaged.

II. References

(1) TURBOMOLE, Ver 7.5.1; TURBOMOLE GmbH, 2021. https://www.turbomole.org/.

(2) TURBOMOLE, Ver 7.7; TURBOMOLE GmbH, 2022. https://www.turbomole.org/.

(3) TURBOMOLE, Ver 7.9; TURBOMOLE GmbH, 2024. https://www.turbomole.org/.

(4) BIOVIA COSMOconfX, Ver 25.0.0; Dassault Systèmes. https://www.3ds.com.

(5) Neese, F. Software Update: The ORCA Program System—Version 5.0. *WIREs Comput. Mol. Sci.* **2022**, *12* (5), e1606. https://doi.org/10.1002/wcms.1606.

(6) BIOVIA COSMOtherm, Ver 25.0.0; Dassault Systèmes, 2025. https://www.3ds.com/products/biovia/cosmo-rs/cosmotherm.

(7) Paenurk, E.; Kaupmees, K.; Himmel, D.; Kütt, A.; Kaljurand, I.; Koppel, I. A.; Krossing, I.; Leito, I. A Unified View to Brønsted Acidity Scales: Do We Need Solvated Protons? *Chem. Sci.* **2017**, *8* (10), 6964–6973. https://doi.org/10.1039/C7SC01424D.

(8) Fifen, J. J.; Dhaouadi, Z.; Nsangou, M. Revision of the Thermodynamics of the Proton in Gas Phase. *J. Phys. Chem. A* **2014**, *118* (46), 11090–11097. https://doi.org/10.1021/jp508968z.

(9) Scholz, F.; Himmel, D.; Eisele, L.; Unkrig, W.; Martens, A.; Schlüter, P.; Krossing, I. The Acidity of the HBr/$AlBr_3$ System: Stabilization of Crystalline Protonated Arenes and Their Acidity in Bromoaluminate Ionic Liquids. *Chem. – Eur. J.* **2015**, *21* (20), 7489–7502. https://doi.org/10.1002/chem.201405952.

(10) Riplinger, C.; Sandhoefer, B.; Hansen, A.; Neese, F. Natural Triple Excitations in Local Coupled Cluster Calculations with Pair Natural Orbitals. *J. Chem. Phys.* **2013**, *139* (13), 134101. https://doi.org/10.1063/1.4821834.

(11) Helgaker, T.; Klopper, W.; Koch, H.; Noga, J. Basis-Set Convergence of Correlated Calculations on Water. *J. Chem. Phys.* **1997**, *106* (23), 9639–9646. https://doi.org/10.1063/1.473863.

(12) Klamt, A. Conductor-like Screening Model for Real Solvents: A New Approach to the Quantitative Calculation of Solvation Phenomena. *J. Phys. Chem.* **1995**, *99* (7), 2224–2235. https://doi.org/10.1021/j100007a062.

(13) Klamt, A.; Jonas, V.; Bürger, T.; Lohrenz, J. C. W. Refinement and Parametrization of COSMO-RS. *J. Phys. Chem. A* **1998**, *102* (26), 5074–5085. https://doi.org/10.1021/jp980017s.

(14) Eckert, F.; Klamt, A. Fast Solvent Screening via Quantum Chemistry: COSMO-RS Approach. *AIChE J.* **2002**, *48* (2), 369–385. https://doi.org/10.1002/aic.690480220.

III. UV-Vis spectra of all compounds used for pK_a measurements

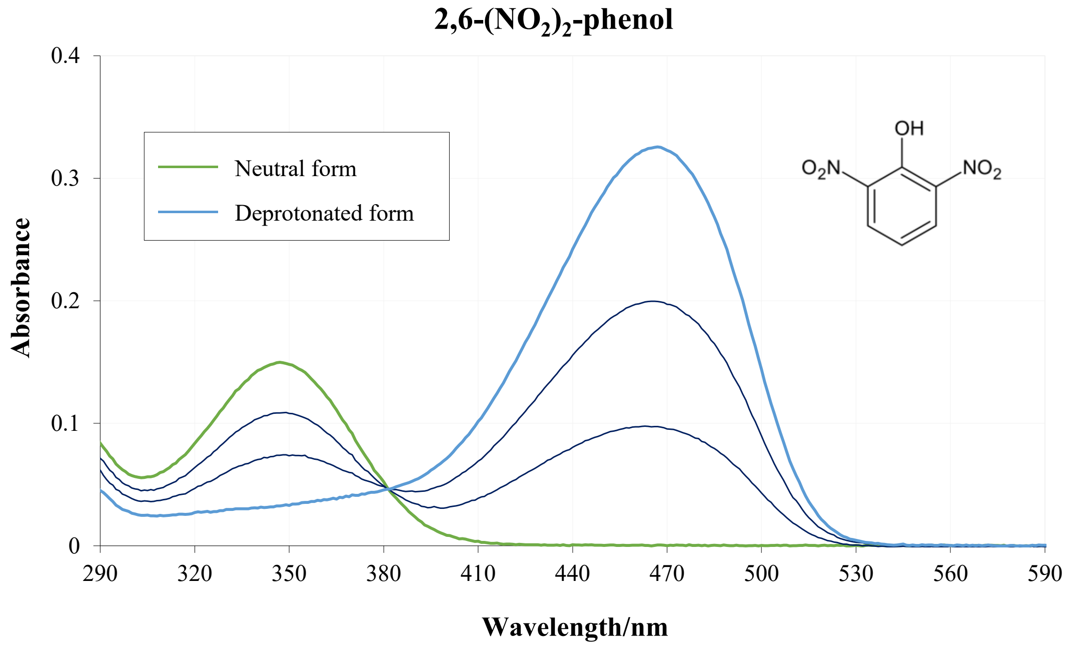

Figure S1. UV-Vis titration spectra of **1** in 1,2-DFB.

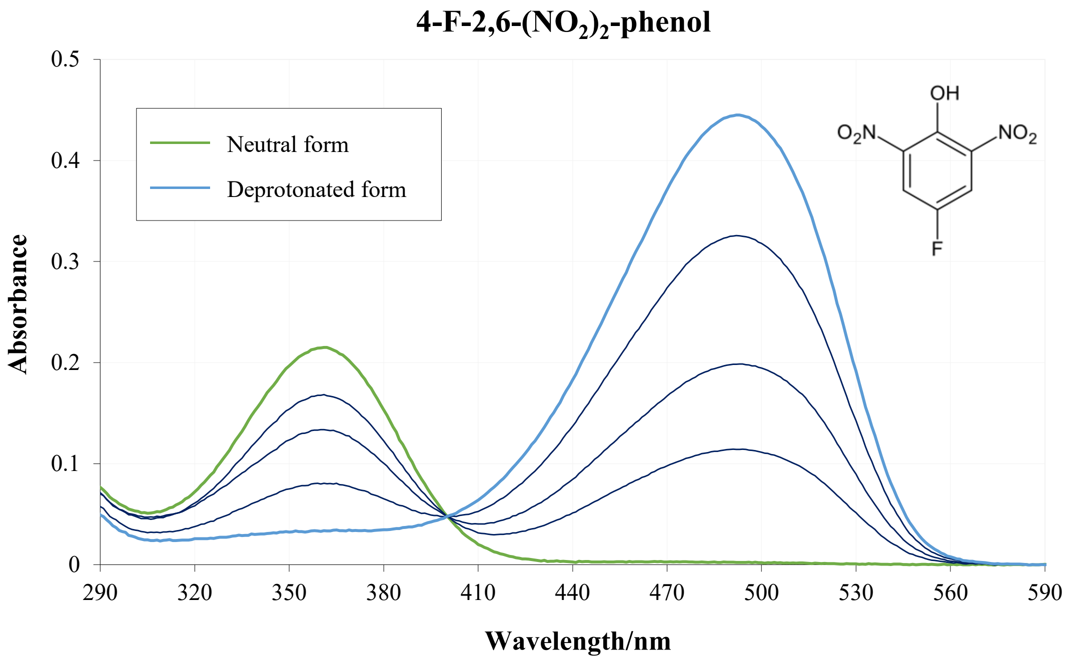

Figure S2. UV-Vis titration spectra of **2** in 1,2-DFB.

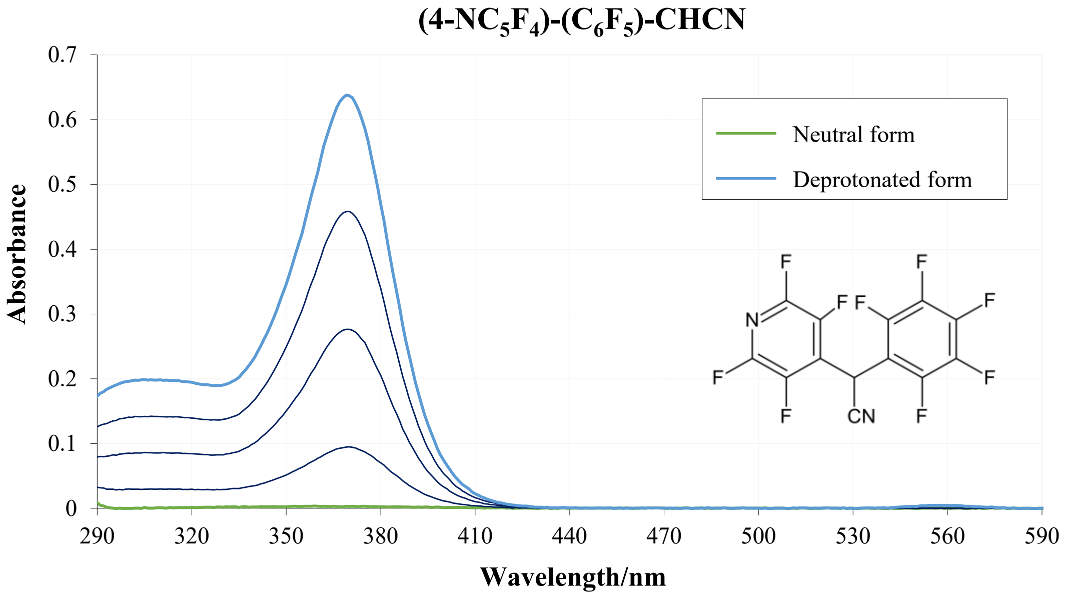

Figure S3. UV-Vis titration spectra of **3** in 1,2-DFB.

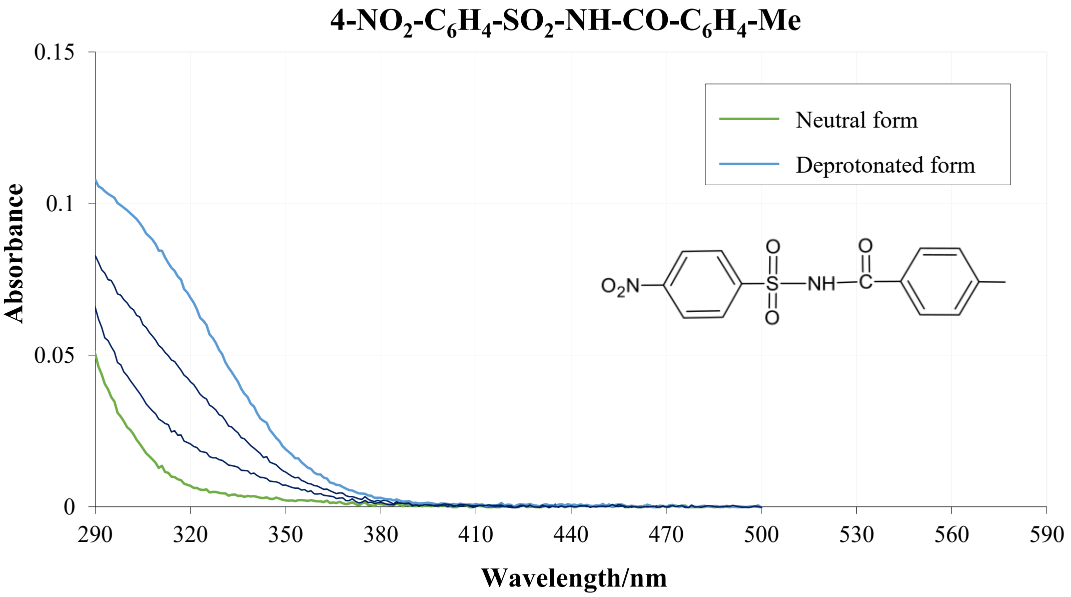

Figure S4. UV-Vis titration spectra of **4** in 1,2-DFB.

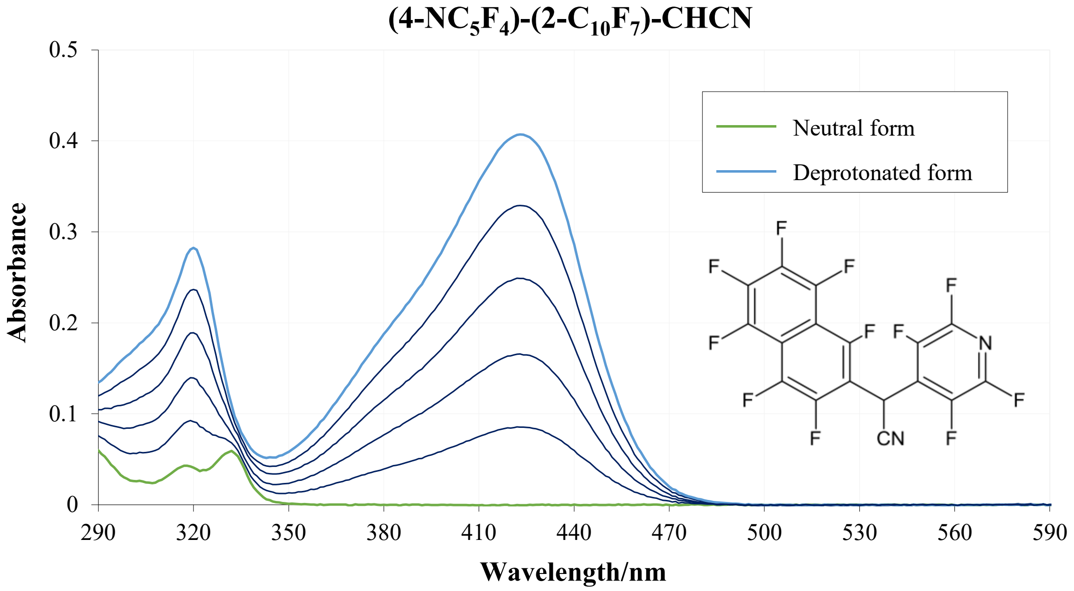

Figure S5. UV-Vis titration spectra of **5** in 1,2-DFB.

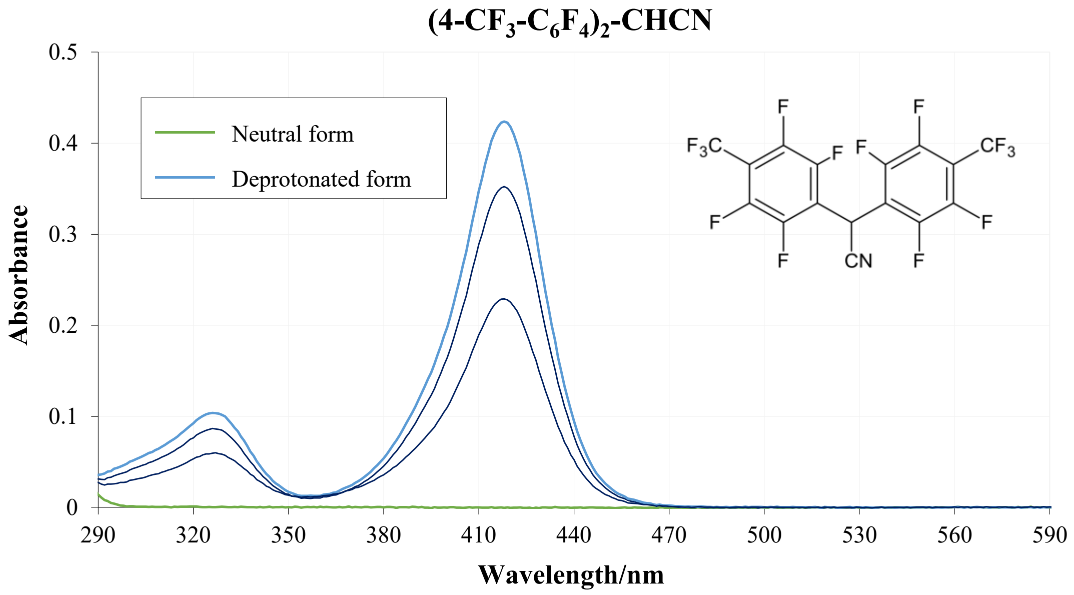

Figure S6. UV-Vis titration spectra of **6** in 1,2-DFB.

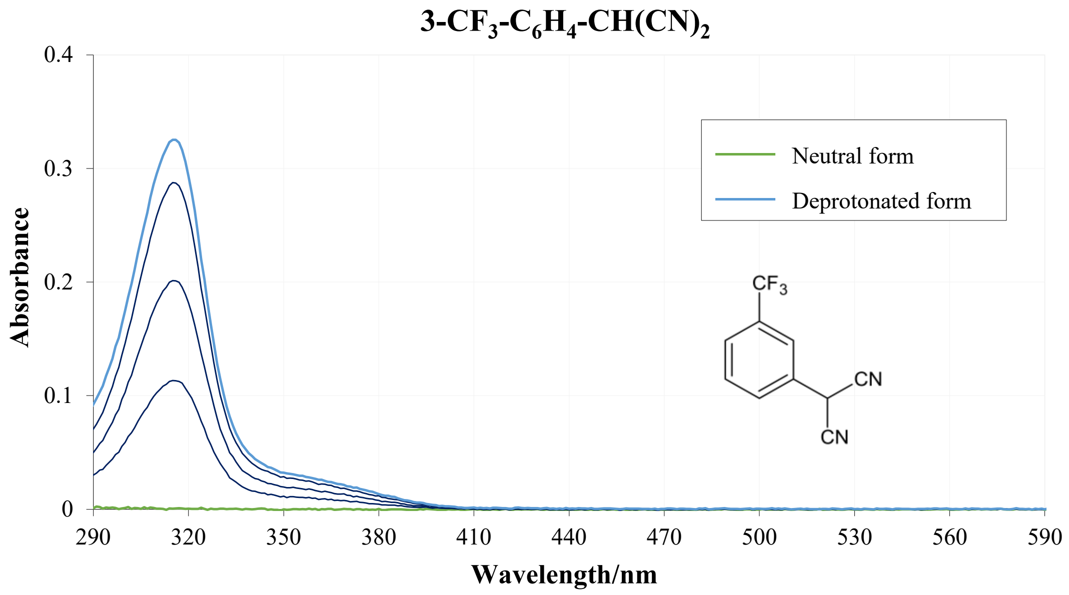

Figure S7. UV-Vis titration spectra of **7** in 1,2-DFB.

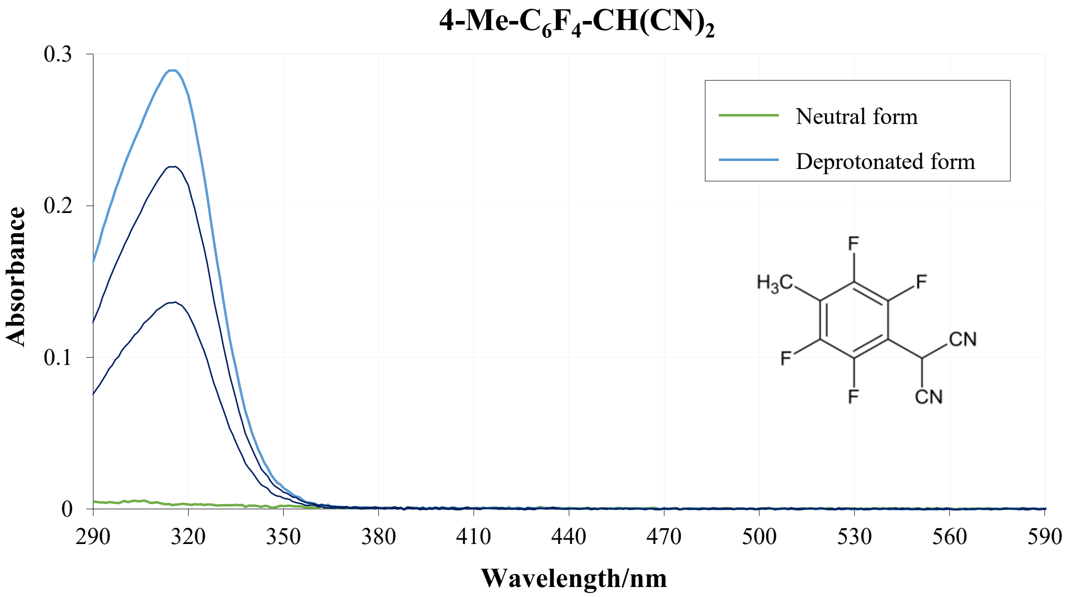

Figure S8. UV-Vis titration spectra of **8** in 1,2-DFB.

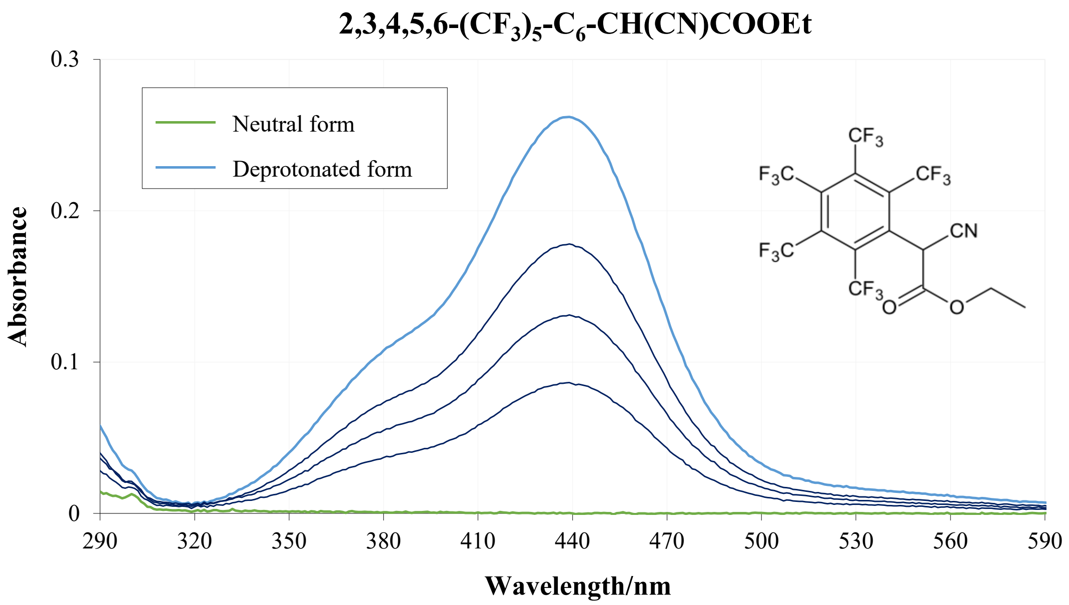

Figure S9. UV-Vis titration spectra of **9** in 1,2-DFB.

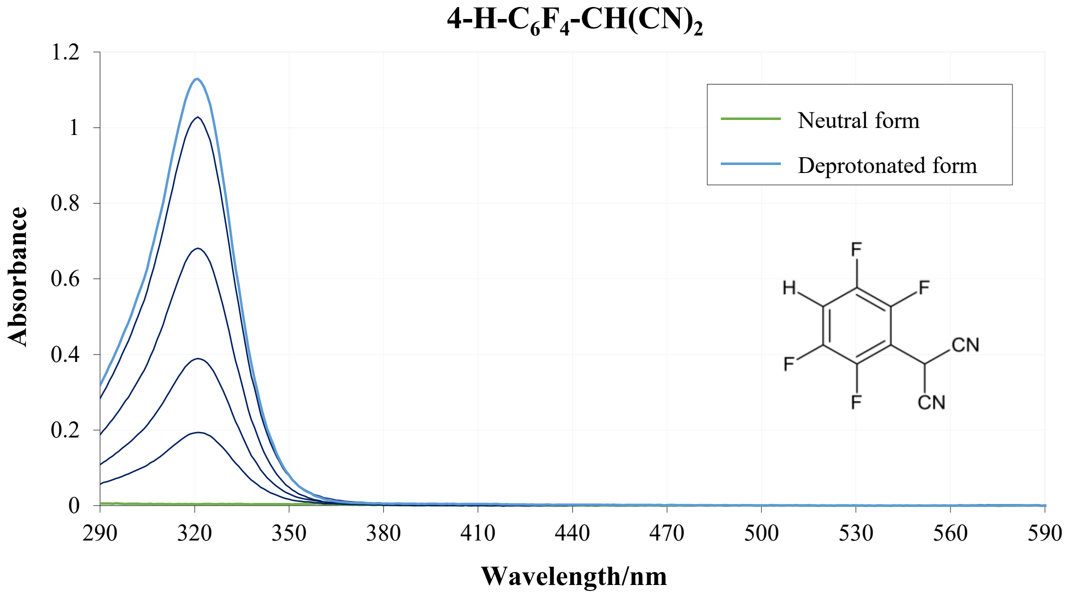

Figure S10. UV-Vis titration spectra of **10** in 1,2-DFB.

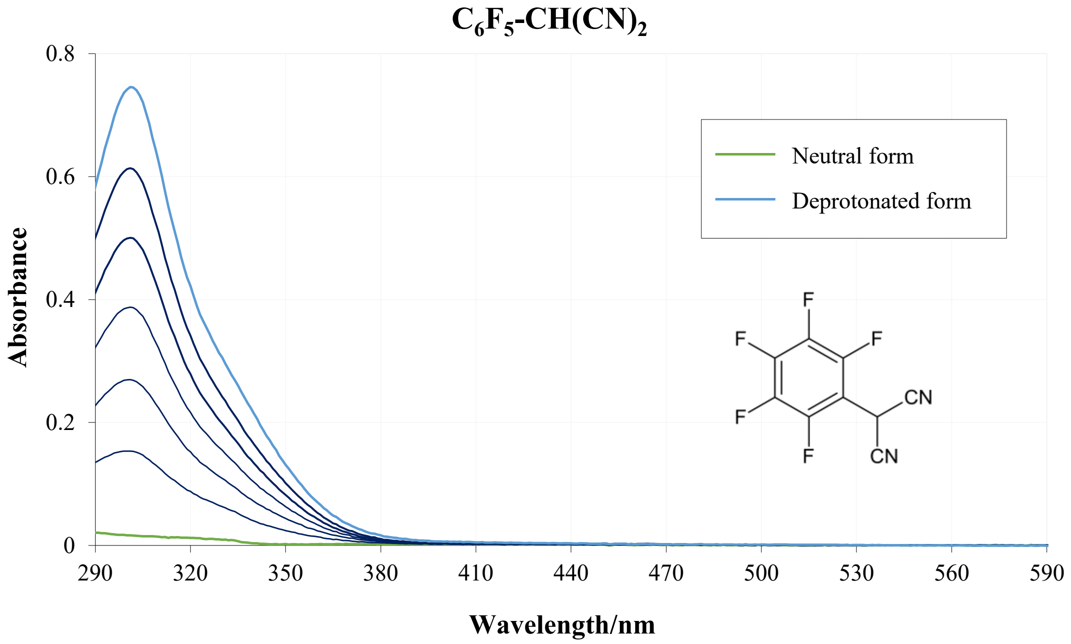

Figure S11. UV-Vis titration spectra of **11** in 1,2-DFB.

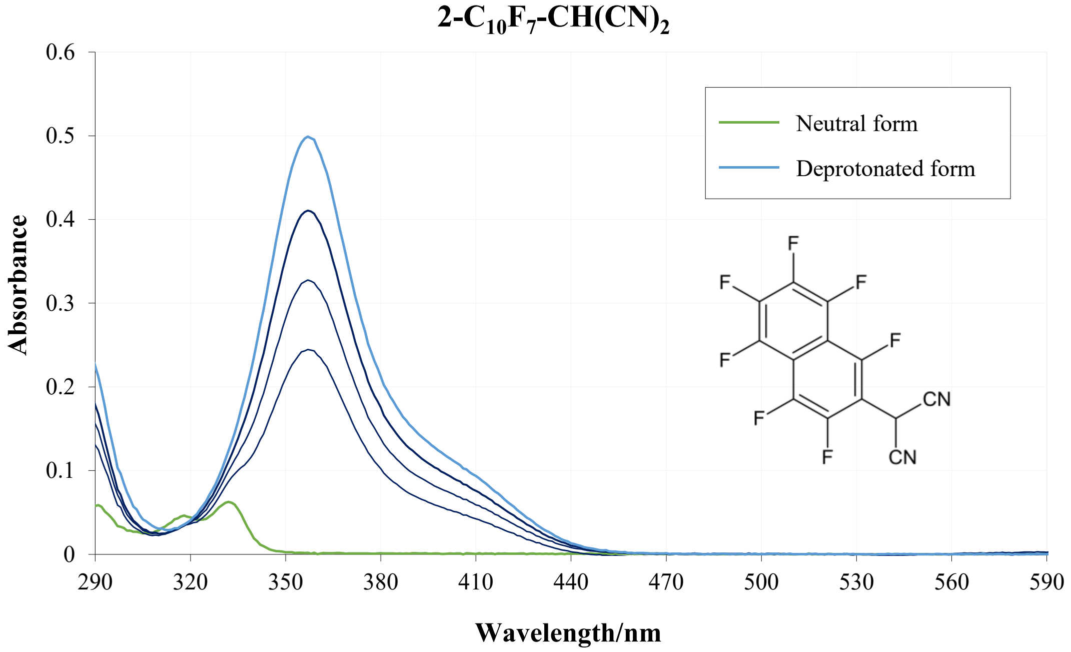

Figure S12. UV-Vis titration spectra of **12** in 1,2-DFB.

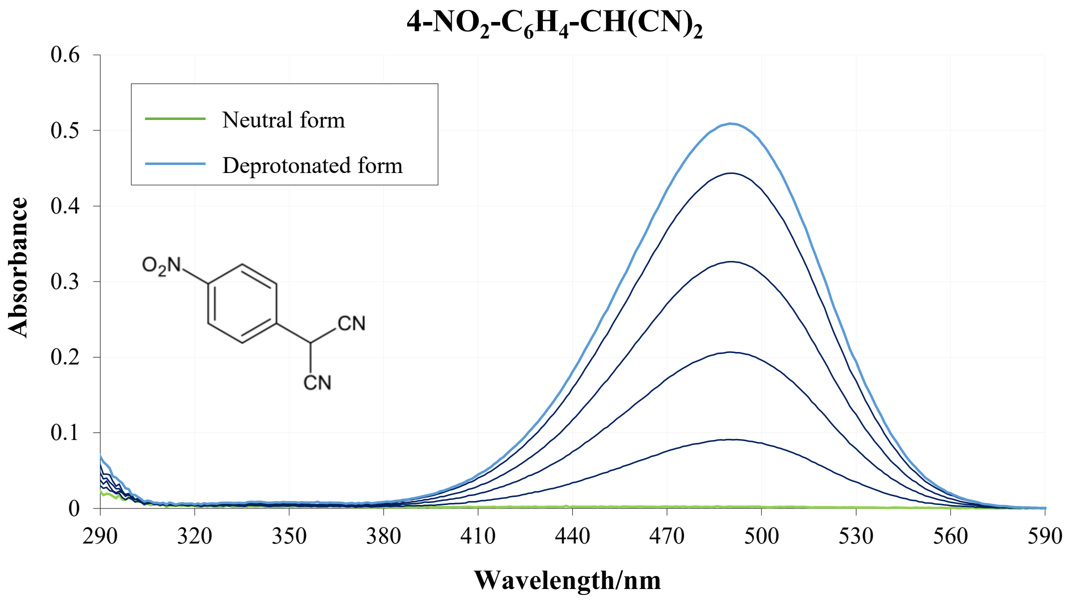

Figure S13. UV-Vis titration spectra of **13** in 1,2-DFB.

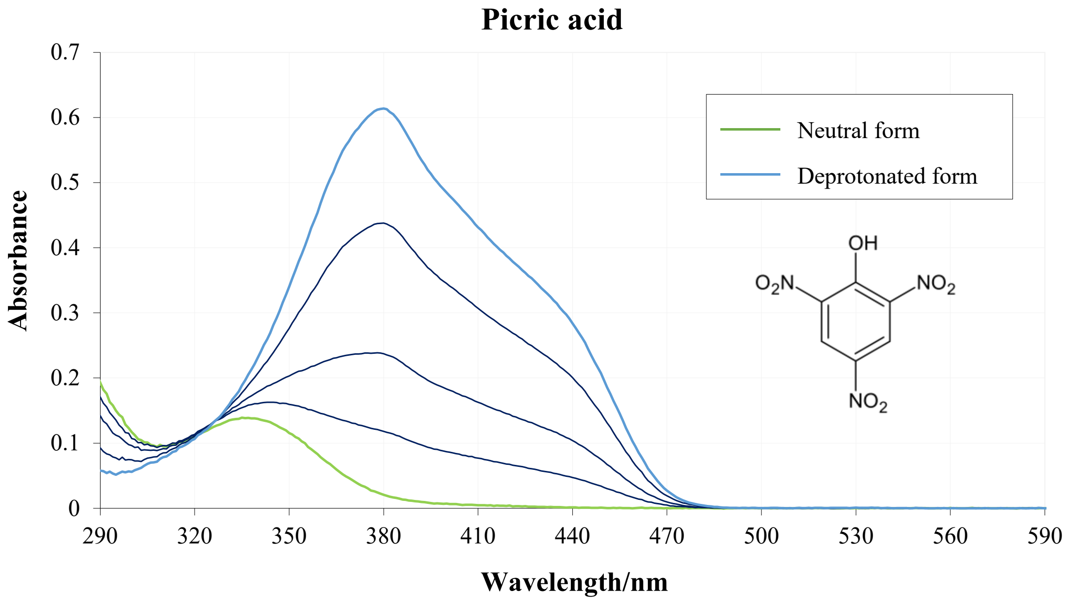

Figure S14. UV-Vis titration spectra of **14** in 1,2-DFB.

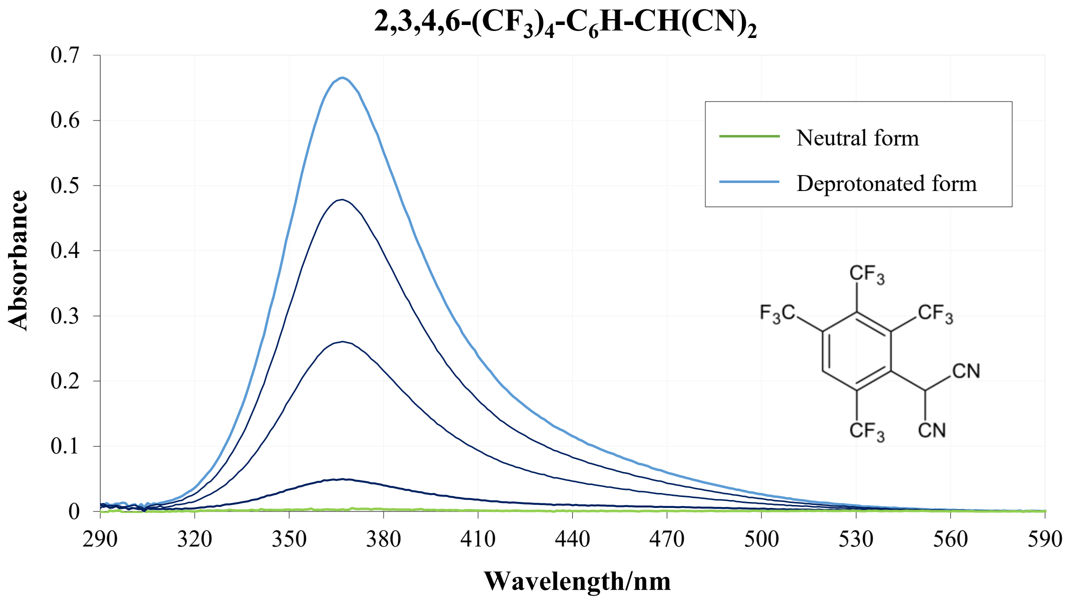

Figure S15. UV-Vis titration spectra of **15** in 1,2-DFB.

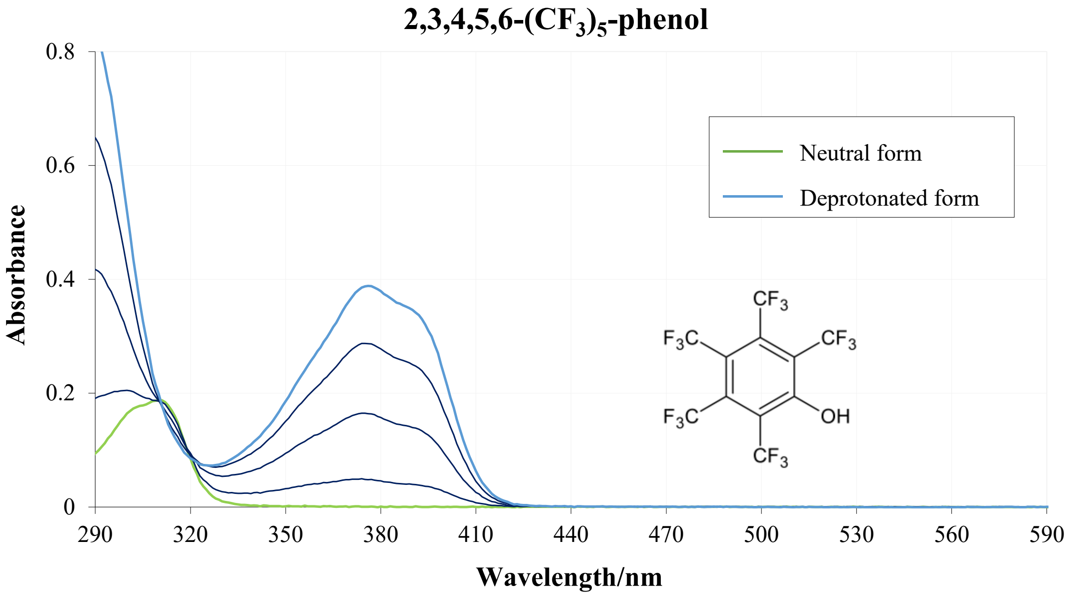

Figure S16. UV-Vis titration spectra of **16** in 1,2-DFB.

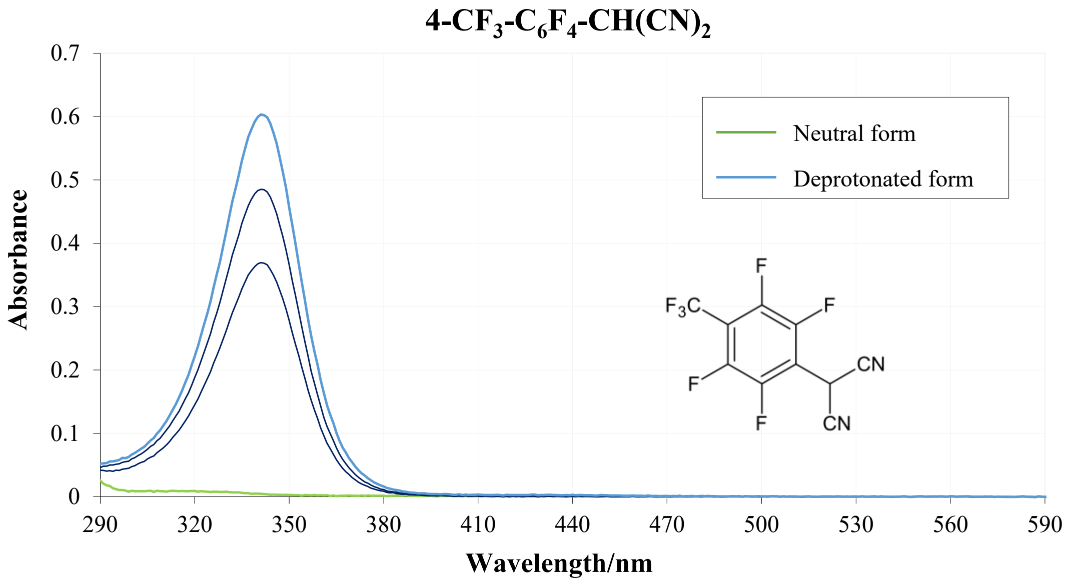

Figure S17. UV-Vis titration spectra of **17** in 1,2-DFB.

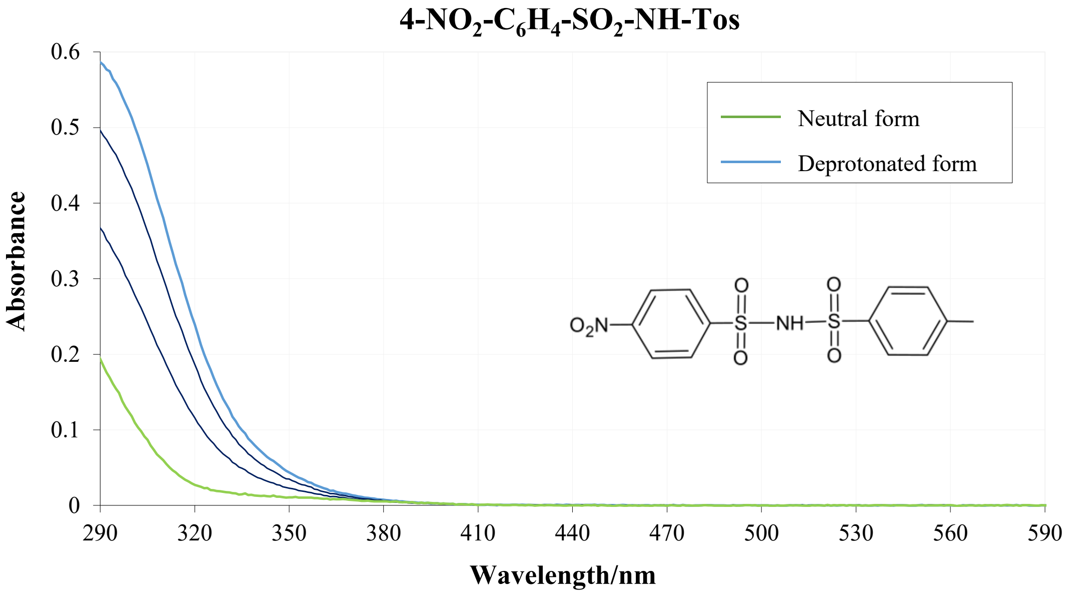

Figure S18. UV-Vis titration spectra of **18** in 1,2-DFB.

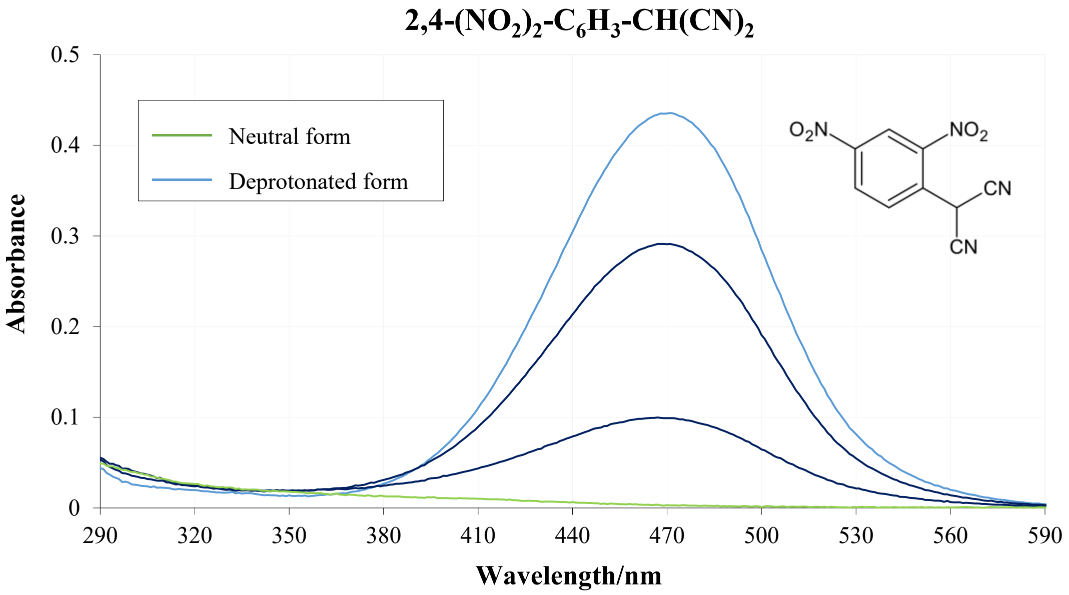

Figure S19. UV-Vis titration spectra of **19** in 1,2-DFB.

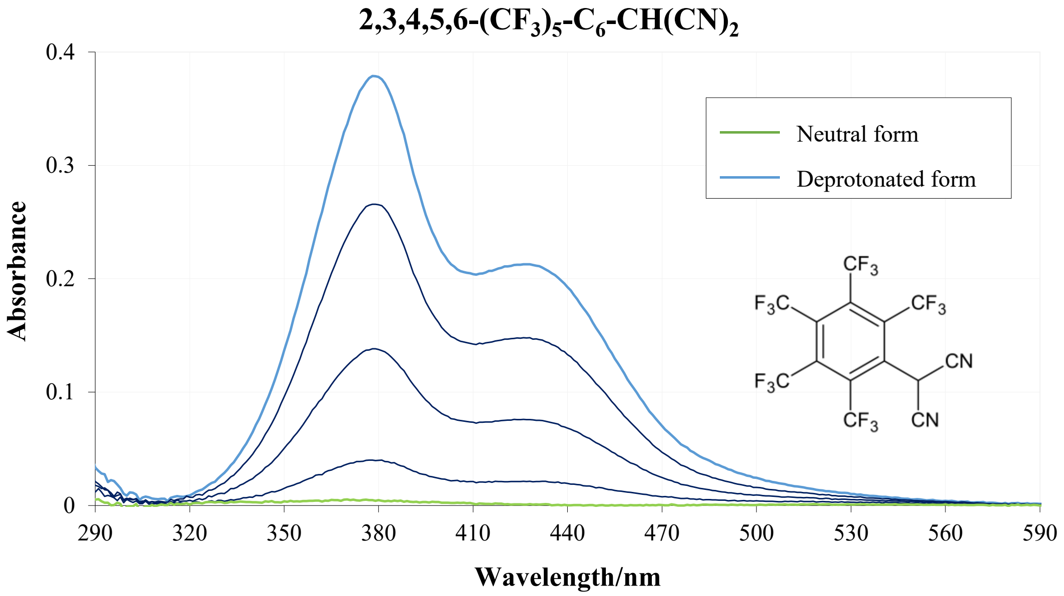

Figure S20. UV-Vis titration spectra of **20** in 1,2-DFB.

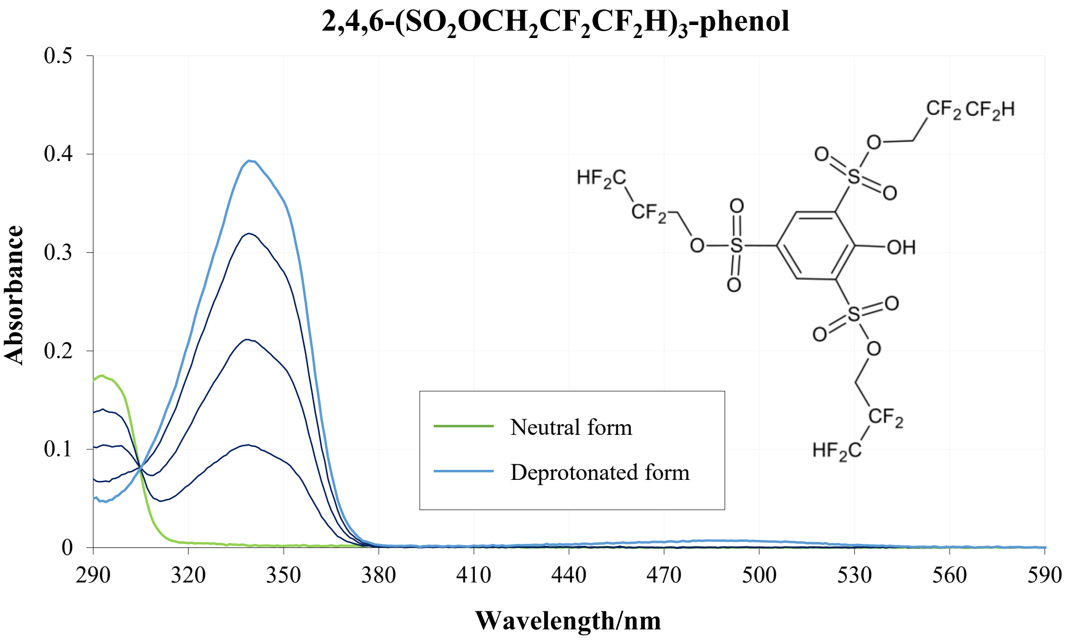

Figure S21. UV-Vis titration spectra of **21** in 1,2-DFB.

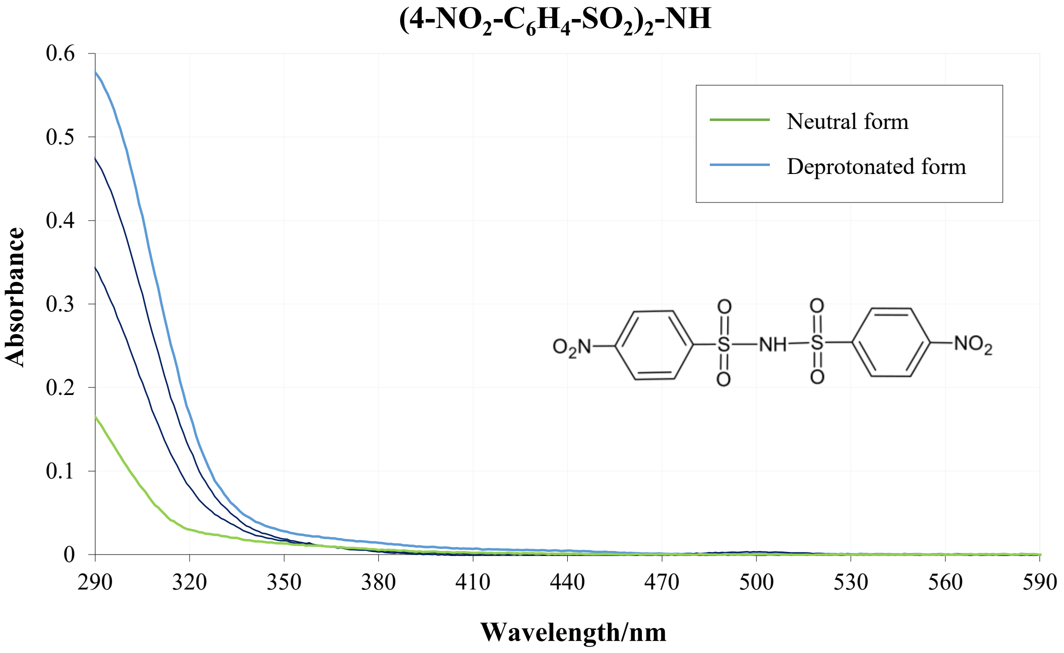

Figure S22. UV-Vis titration spectra of **22** in 1,2-DFB.

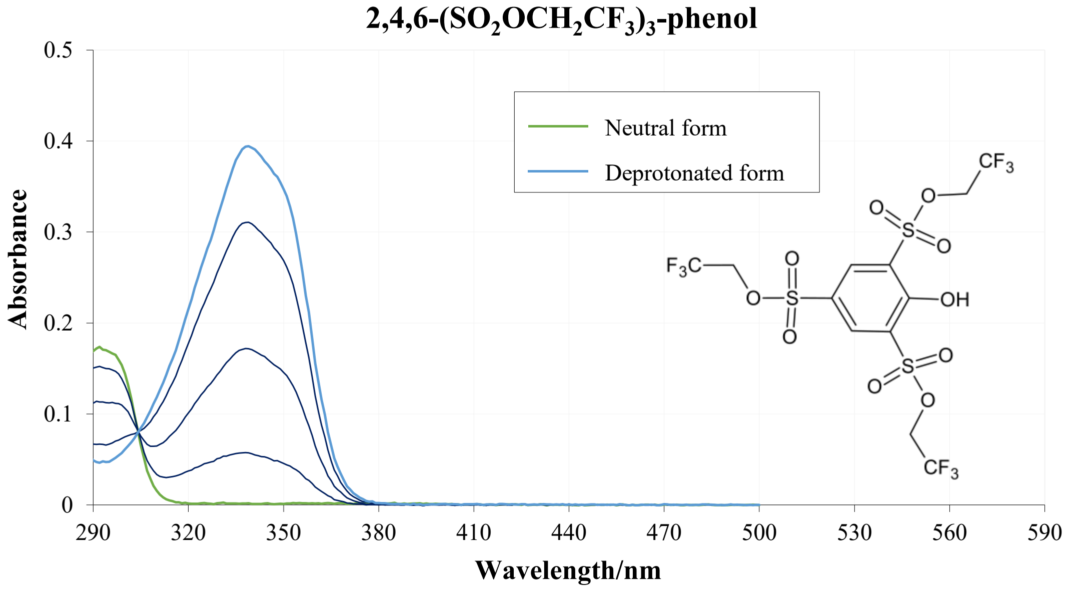

Figure S23. UV-Vis titration spectra of **23** in 1,2-DFB.

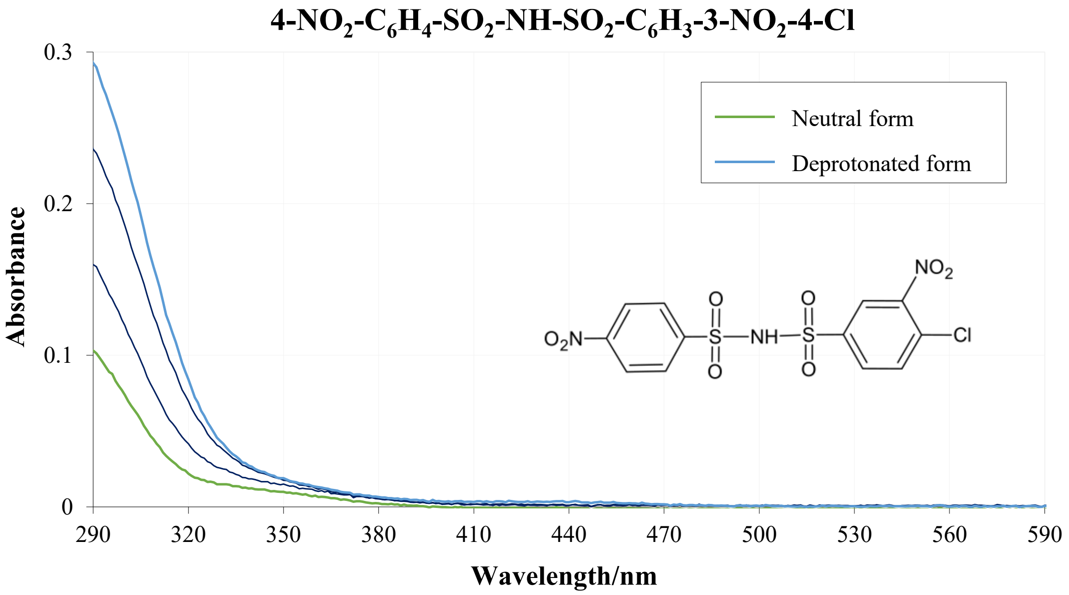

Figure S24. UV-Vis titration spectra of **24** in 1,2-DFB.

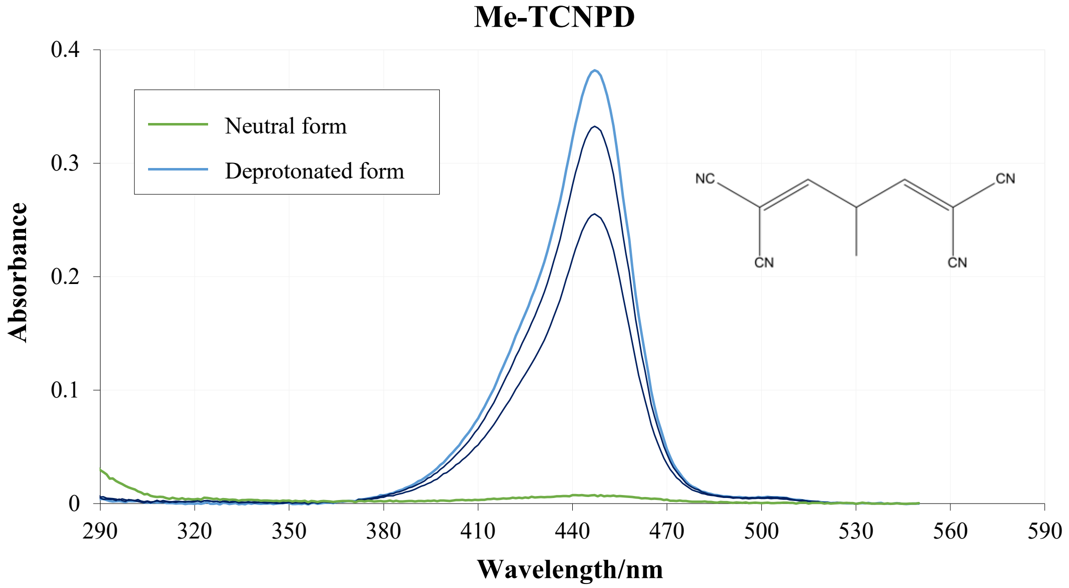

Figure S25. UV-Vis titration spectra of **25** in 1,2-DFB.

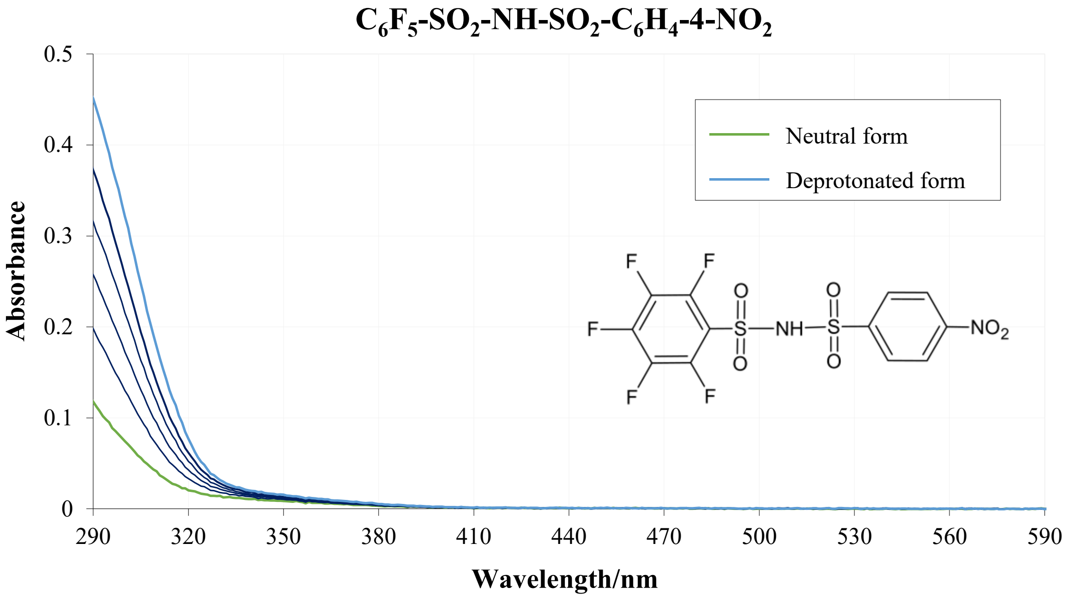

Figure S26. UV-Vis titration spectra of **26** in 1,2-DFB.

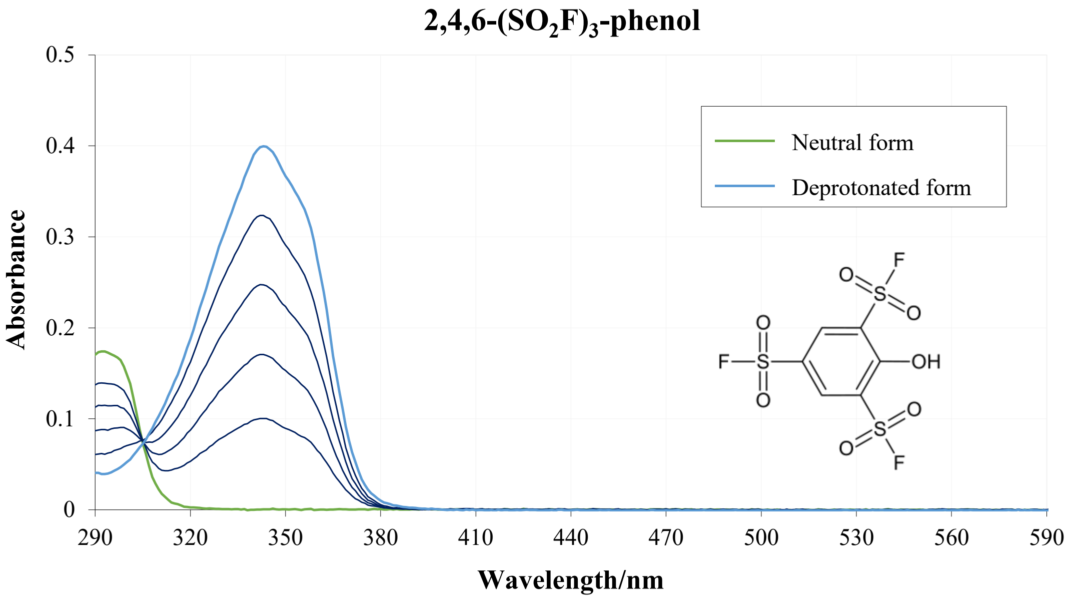

Figure S27. UV-Vis titration spectra of **27** in 1,2-DFB.

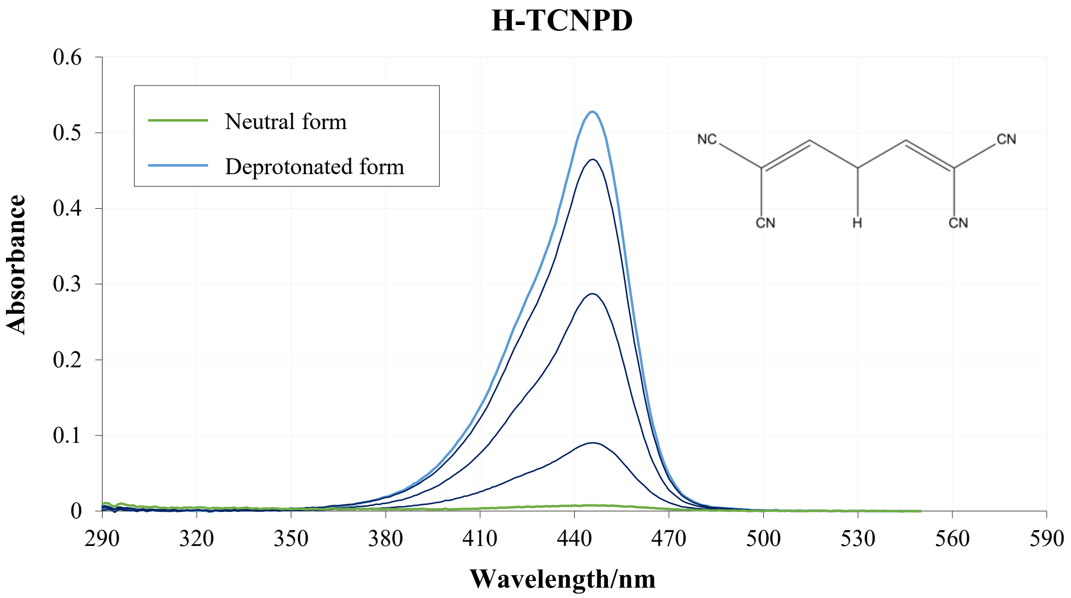

Figure S28. UV-Vis titration spectra of **28** in 1,2-DFB.

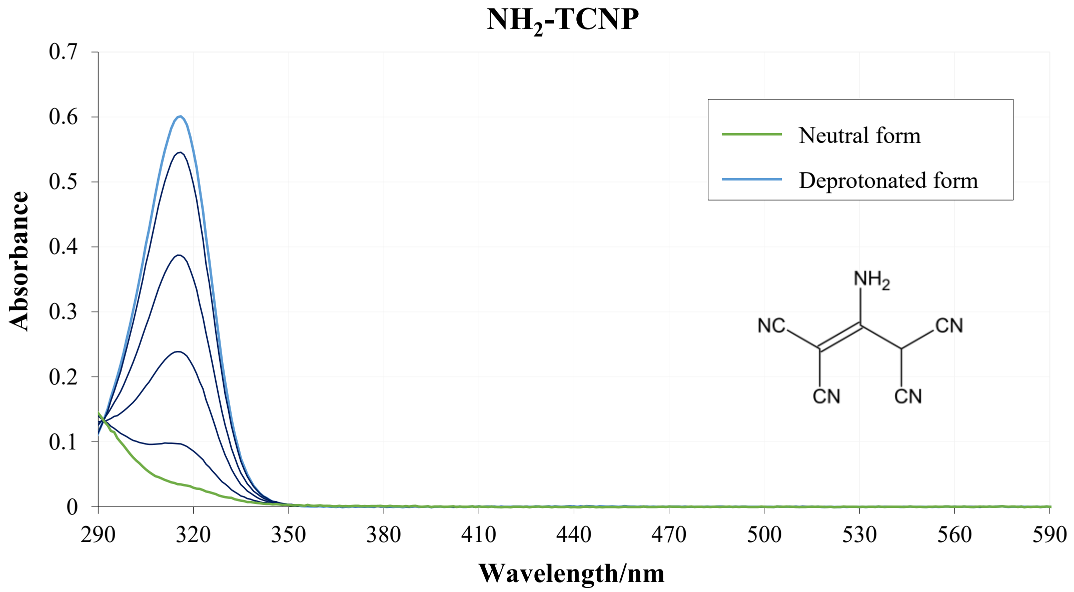

Figure S29. UV-Vis titration spectra of **29** in 1,2-DFB.

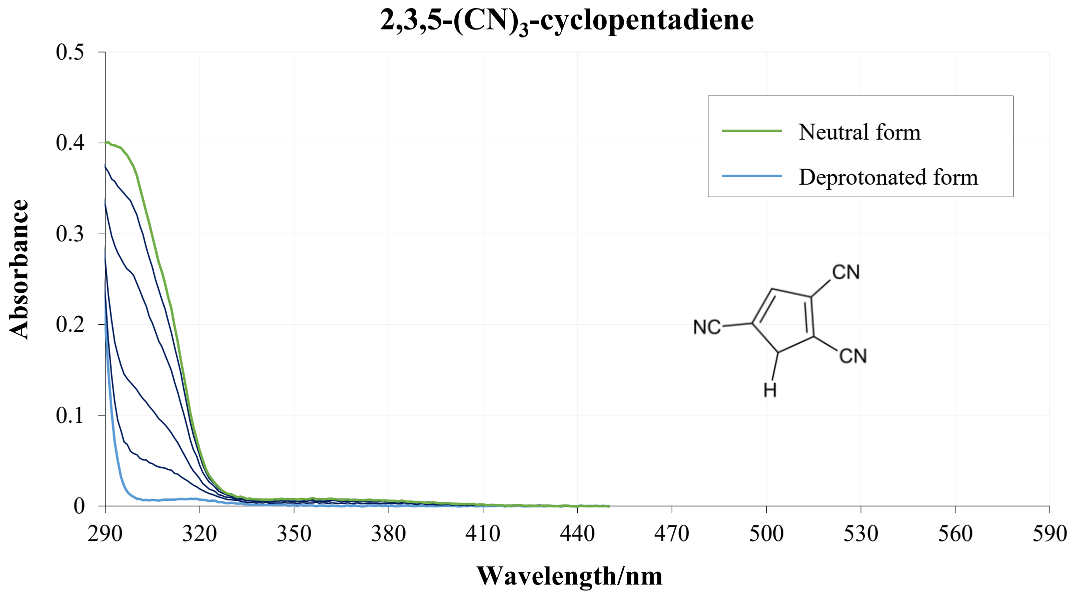

Figure S30. UV-Vis titration spectra of **30** in 1,2-DFB.

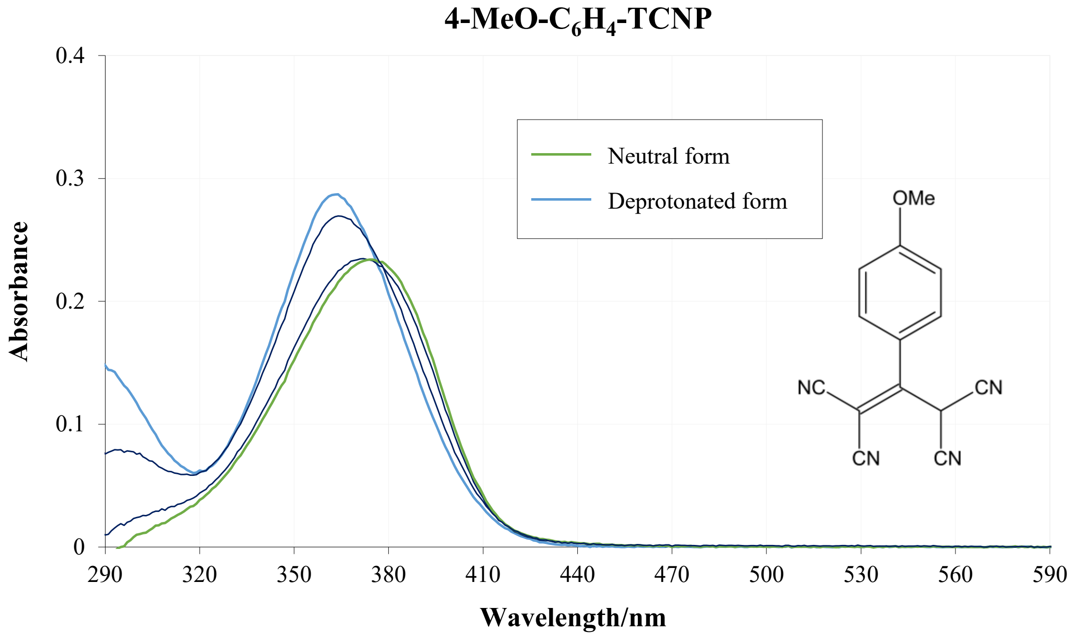

Figure S31. UV-Vis titration spectra of **31** in 1,2-DFB.

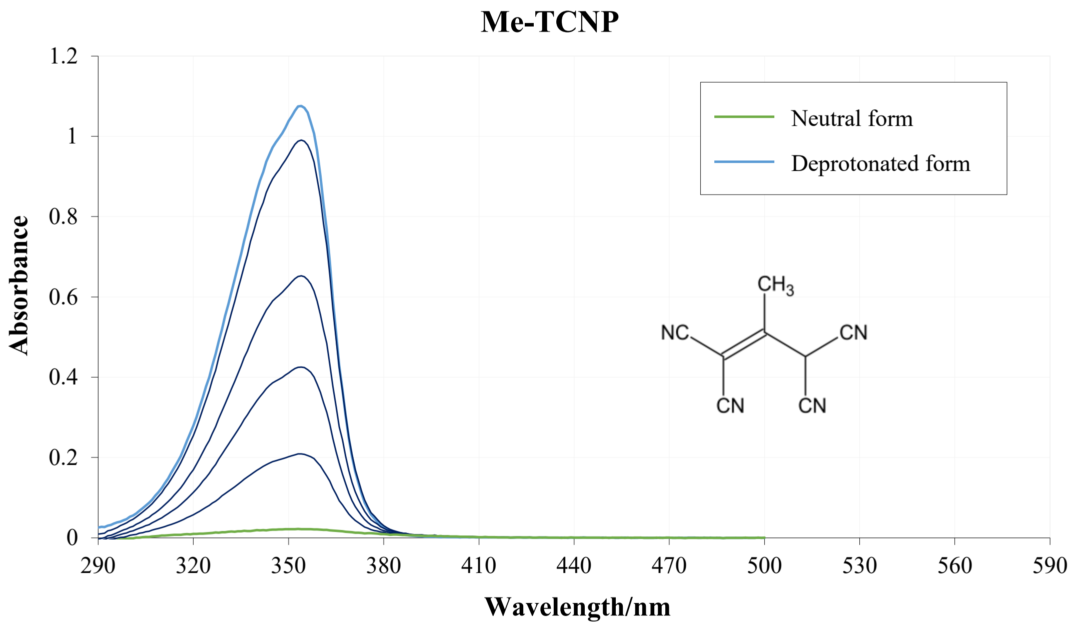

Figure S32. UV-Vis titration spectra of **32** in 1,2-DFB.

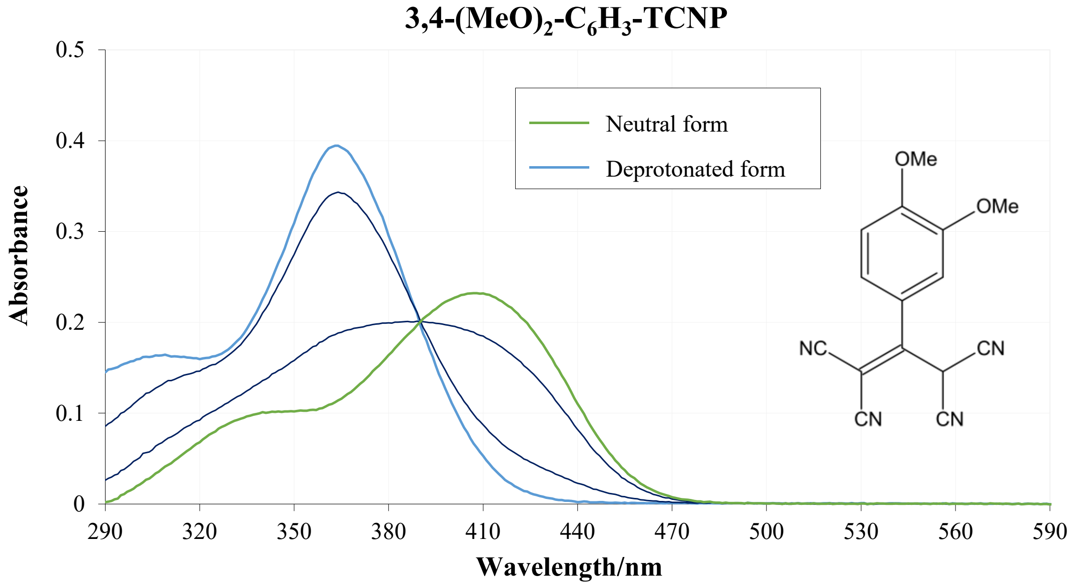

Figure S33. UV-Vis titration spectra of **33** in 1,2-DFB.
